# Phase separation drives NLRP3 inflammasome activation independently of Golgi localization

**DOI:** 10.1038/s41419-026-09059-4

**Published:** 2026-07-06

**Authors:** Yuansen Zhu, Chenchao Wei, Xiang Yin, Bolong Lin, Xiaqiong Wang, Hongbin He, Wei Jiang, Rongbin Zhou

**Affiliations:** State Key Laboratory of Immune Response and Immunotherapy, Center for Advanced Interdisciplinary Science and Biomedicine of IHM, School of Basic Medical Sciences, Division of Life Sciences and Medicine, University of Science and Technology of China, Hefei, China

**Keywords:** Cell death, Cell death and immune response

## Abstract

The NLRP3 inflammasome plays a pivotal role in sterile inflammation and various diseases, yet the mechanisms underlying its activation remain elusive. Previous studies have implicated both NLRP3 Golgi localization and palmitoylation-mediated phase separation in its activation; however, mutations in palmitoylation sites or the FISNA domain’s intrinsically disordered region (IDR) concurrently disrupt both processes, hindering delineation of their individual contributions to inflammasome activation. Here, we show that NLRP3 exhibits phase separation and Golgi localization simultaneously during inflammasome activation. The polybasic region and FISNA IDR each mediate both processes, whereas lipid-binding motif deletion abolishes Golgi localization without compromising phase separation or inflammasome activity. Conversely, the C-terminal IDR is essential for phase separation and inflammasome activity but dispensable for Golgi localization. By mechanistically uncoupling these processes, our findings establish that phase separation drives NLRP3 activation independently of Golgi localization, thus redefining the spatial and biophysical paradigm of inflammasome assembly.

## Introduction

Sterile inflammation, triggered by damage-associated molecular patterns (DAMPs), drives the pathogenesis of numerous human diseases, including gout, atherosclerosis, Alzheimer’s disease, and type 2 diabetes [1, 2]. At the core of this process lies the NLRP3 inflammasome, a multiprotein complex that detects diverse cellular stressors and activates caspase-1, thereby promoting the maturation and secretion of pro-inflammatory cytokines such as interleukin-1β (IL-1β) and IL-18, alongside pyroptotic cell death. Despite its profound clinical implications, the molecular mechanisms governing NLRP3 activation remain incompletely elucidated.

Prior research has linked mitochondrial dysfunction[3], potassium (K^+^) efflux [4, 5], and lysosomal membrane permeabilization (LMP) to NLRP3 activation, yet the post-sensing alterations in NLRP3 itself—particularly its spatial and biophysical prerequisites for inflammasome assembly—are poorly defined. Early studies underscored the binding of NLRP3 to phosphatidylinositol-4-phosphate (PI4P) and its translocation to the Golgi apparatus (often referred to interchangeably in this context as trans-Golgi network (TGN) localization, membrane association, or vesicular distribution) as pivotal for activation [6–8]. However, recent findings challenge this view: certain human NLRP3 mutants aggregate and activate independently of TGN localization [9–12]. Furthermore, a recent report posits that palmitoylation-dependent liquid-liquid phase separation (LLPS)—wherein proteins condense into membraneless compartments—serves as the primary mechanism for NLRP3 oligomerization and activation [11]. Nonetheless, disentangling these processes has proven challenging, as mutations targeting palmitoylation sites or the IDR within the FISNA domain disrupt both Golgi localization and phase separation [11], obscuring their distinct roles.

In this study, we dissect the roles of phase separation and Golgi localization in NLRP3 inflammasome activation through targeted mutagenesis and functional assays. We show that NLRP3 exhibits both phase separation and Golgi localization during activation, with the polybasic region proving essential for both phenomena. The FISNA IDR facilitates these dual processes, whereas deletion of the lipid-binding motif eliminates Golgi targeting without compromising inflammasome activity. Conversely, the C-terminal IDR is indispensable for activity but dispensable for Golgi association. These findings establish that phase separation propels NLRP3 activation independently of Golgi localization, redefining inflammasome regulatory paradigms and unveiling prospects for precision therapies in inflammatory disorders.

## Results

### NLRP3 undergoes phase separation and Golgi localization during inflammasome activation

**Fig. 1:**
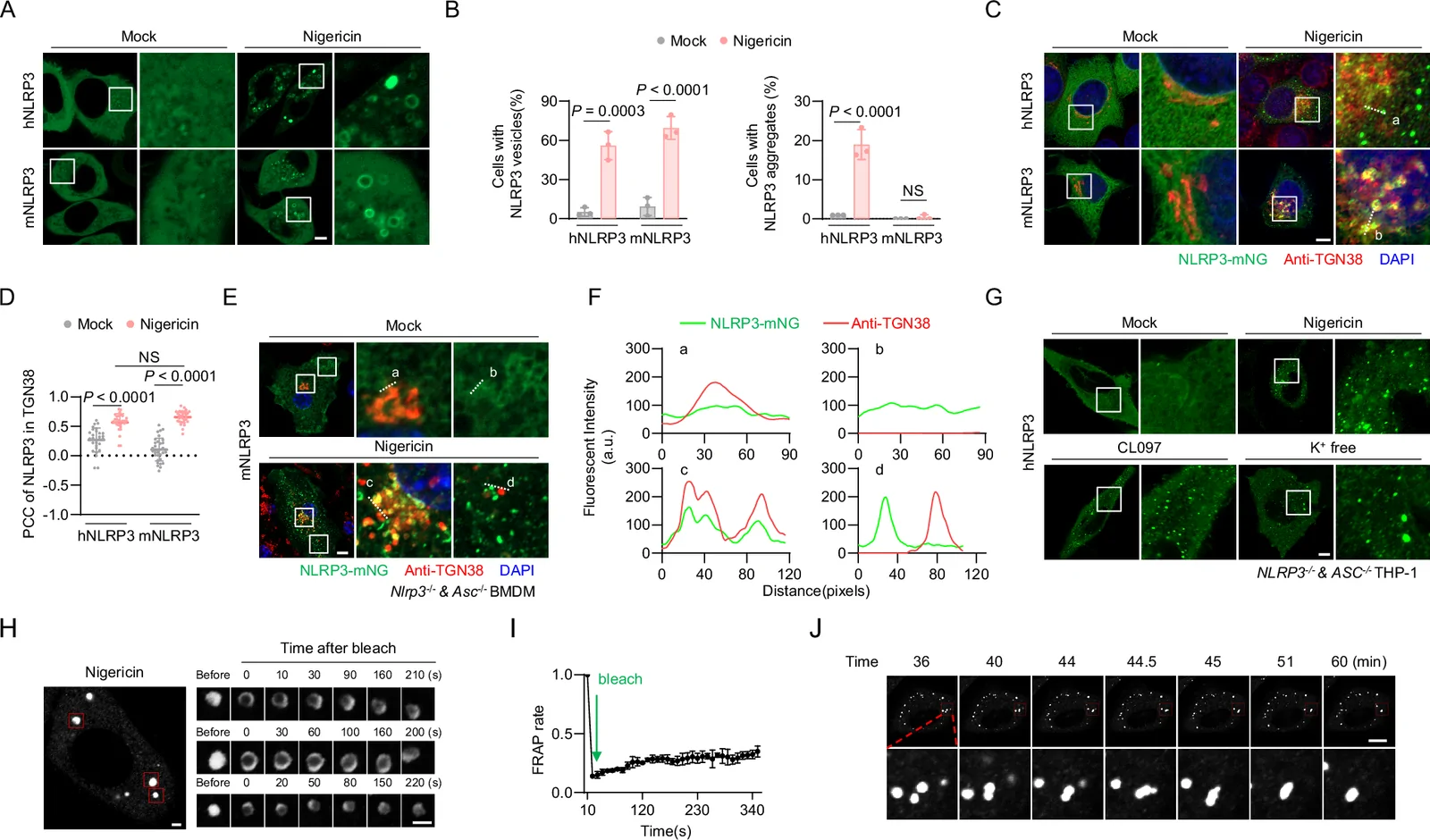
NLRP3 exists in both vesicles and aggregates during activation. **A** Representative images of HeLa cells stably expressing human or mouse NLRP3 stimulated with 10 μM nigericin for 60 min. Scale bar, 5 μm. **B** Quantification of cells containing NLRP3 vesicles or aggregates (>100 cells per group, *n* = 3). **C** Representative images showing colocalization of TGN and NLRP3 in HeLa cells stably expressing human or mouse NLRP3–mNG before and after nigericin stimulation. Scale bar, 5 μm. **D** Pearson’s correlation coefficient (PCC) analysis of NLRP3–TGN38 colocalization (10 cells per group, *n* = 3). **E**, **F** Representative images showing colocalization of the TGN and NLRP3 in mNLRP3–mNeonGreen reconstituted *Nlrp3*^*−*/−^ and *Asc*^−/−^ double-knockout BMDMs, before and after nigericin stimulation. Scale bar, 5 μm. **F** Line-scan profiles showing normalized mean fluorescence intensity (MFI) of NLRP3 and TGN signals in (**E**). **G** Representative images of stably expressing human NLRP3 in *NLRP3*^*−*/−^ and *ASC*^−/−^ double-knockout THP-1 cells treated with 10 μM nigericin (60 min), 40 μM CL097 (60 min), or K^+^-free medium (60 min). Scale bar, 5 μm. **H**, **I** FRAP analysis of human NLRP3–mNG in HeLa cells after 60 min of nigericin stimulation. Bleached regions are indicated (red boxes). Scale bar, 2 μm. **J** Time-lapse imaging of NLRP3 droplet fusion in HeLa cells 60 min after nigericin stimulation. Scale bar, 5 μm. Data are presented as mean ± SD. In all quantifications, *n* represents the number of independent experiments. Data shown in (**A**, **C**, **E**, **G**, **H**, **J**) are representative of three independent experiments. Images in (**A**, **C**) are quantified in (**B**, **D**), respectively. Significance determined by two-way ANOVA followed by Tukey’s multiple comparisons test (**B**, **D**); NS not significant.

To track the dynamic changes during NLRP3 activation, stable HeLa cell lines expressing either human or mouse NLRP3 fused to mNeonGreen were generated. Upon stimulation with nigericin for 1 h, mouse NLRP3 exhibited a vesicular enrichment pattern, whereas human NLRP3 displayed both vesicular distribution and punctate aggregation (Fig. 1A, B). Consistent with previous findings [6, 8, 13], TGN staining revealed substantial co-localization of both mouse and human NLRP3 with the TGN (Figs. 1C, D and S1A). In contrast, the punctate aggregates of human NLRP3 were entirely independent of the TGN (Figs. 1C, D and S1A), suggesting the presence of distinct subcellular populations of NLRP3 following stimulation.

Given that HeLa cells provide a human cellular context, we hypothesized that heterologous expression of murine NLRP3 might not faithfully recapitulate its physiological distribution. To test this, mouse NLRP3–mNeonGreen was stably expressed via retroviral transduction in primary bone marrow-derived macrophages (BMDMs) from *Nlrp3*^*−*/−^ and *Asc*^*−*^^/^^*−*^ double-knockout mice. Under these physiologically relevant conditions, stimulated mouse NLRP3 exhibited localization patterns closely resembling those of human NLRP3 in HeLa cells, displaying both TGN association and prominent droplet-like condensates (Fig. 1E, F). These findings indicate that the previously unobserved phase separation behavior of mouse NLRP3 likely resulted from an inappropriate heterologous cellular microenvironment.

To determine whether NLRP3 condensation represents a general feature of inflammasome activation, two independent activation paradigms were employed: CL097 stimulation, which activates NLRP3 independently of K^+^ efflux [14], and K^+^-free medium treatment, which induces activation through intracellular K^+^ depletion [15]. Both treatments consistently triggered aggregate formation of human NLRP3 in HeLa and THP-1 cells (Figs. S1B and 1G), establishing aggregation as a conserved molecular response across distinct activation pathways. Notably, neither stimulus induced appreciable NLRP3 co-localization with the TGN (Fig. S1B, C), indicating that TGN association is a stimulus-specific rather than a universal prerequisite for NLRP3 activation.

We next investigated the biophysical properties of NLRP3 aggregates. Previous studies have shown that NLRP6, another NOD-like receptor family member, undergoes LLPS through direct binding to double-stranded RNA [16]. Recent evidence also suggests that human NLRP3 forms LLPS-like assemblies [11]. To determine whether these structures represent bona fide LLPS-driven condensates, fluorescence recovery after photobleaching (FRAP) analysis was performed on NLRP3 aggregates in HeLa cells stably expressing human NLRP3 following nigericin stimulation. Quantitative measurements revealed progressive fluorescence recovery within the bleached regions over time (Fig. 1H, I), indicating dynamic molecular exchange within the aggregates. Supporting their liquid-like nature, live-cell imaging captured continuous fusion events between adjacent NLRP3 droplets during 60 min of nigericin stimulation (Fig. 1J and Movie S1). Similar liquid-droplet behavior was consistently observed under alternative activation conditions, with CL097 stimulation or K^+^-free treatment generating NLRP3 aggregates that exhibited intra-droplet fluidity and inter-droplet fusion (Fig. S1D–I and Movies S2 and S3). Notably, fluorescence recovery kinetics inversely correlated with droplet size (Figs. 1H and S1D, G), suggesting a gradual maturation of NLRP3 condensates from liquid-like to more solid-like states.

### The polybasic region of NLRP3 is essential for both phase separation and Golgi localization

**Fig. 2:**
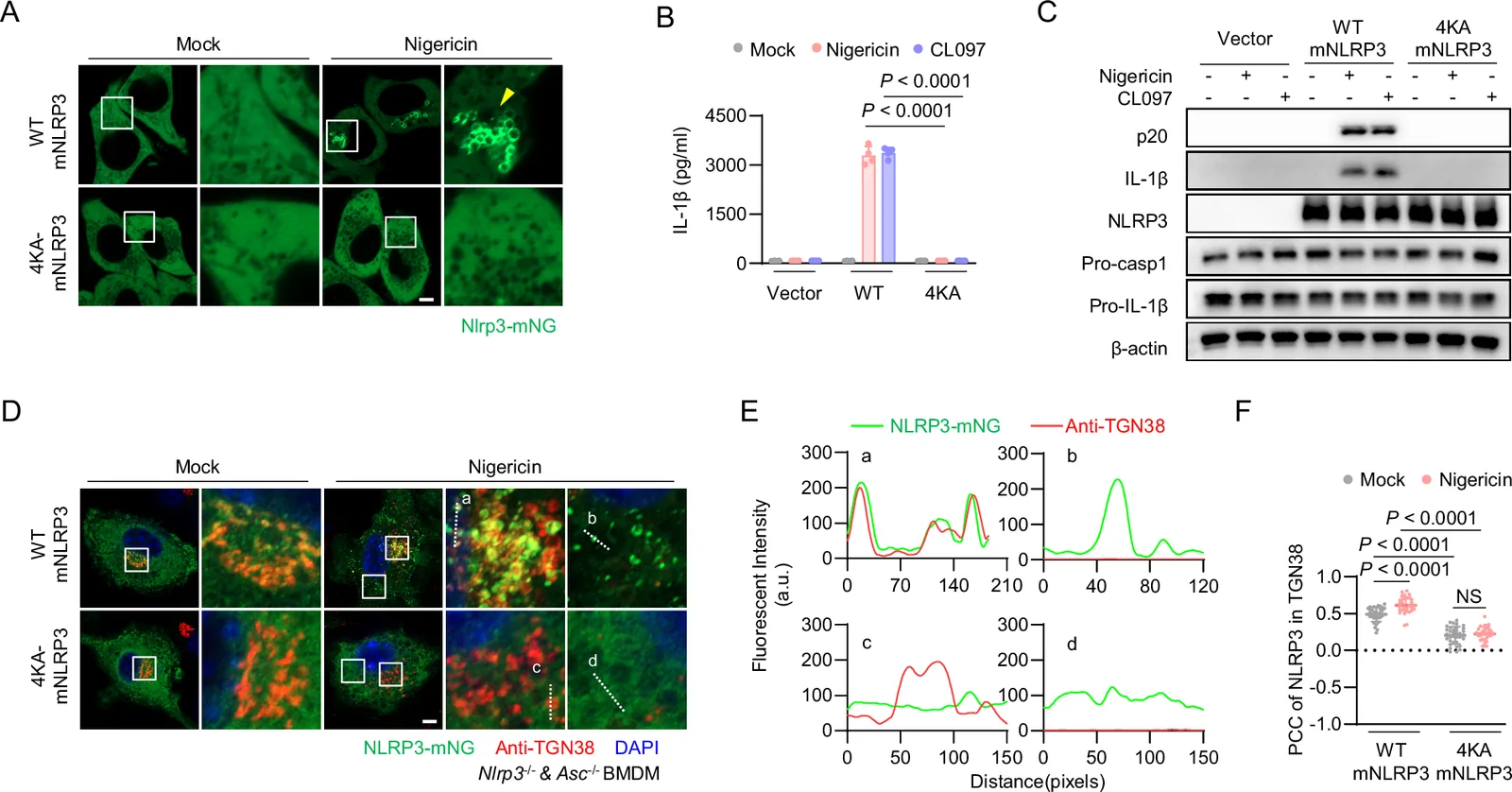
Role of the polybasic region of NLRP3 in phase separation and Golgi localization. **A** Representative images of HeLa cells stably expressing wild-type or 4KA mutant mouse NLRP3 before and after 10 μM nigericin stimulation for 60 min. Scale bar, 5 μm. **B**, **C** *Nlrp3*^*−/−*^ BMDMs complemented with wild-type or 4KA mutant mouse NLRP3 were stimulated with 10 μM nigericin or 40 μM CL097 for 60 min. **B** IL-1β levels in supernatants measured by ELISA (*n* = 3). **C** Western blot analysis of cleaved caspase-1 (p20) and IL-1β in supernatants, and NLRP3, pro–caspase-1, pro–IL-1β, and β-actin in cell lysates. **D–F** *Nlrp3*^*−*^^/^^*−*^ and *Asc*^*−*^^/^^*−*^ double-knockout BMDMs stably expressing wild-type or 4KA mutant mouse NLRP3 were analyzed for colocalization with the TGN before and after nigericin stimulation. **D** Representative confocal images. Scale bar, 5 μm. **E** Line-scan profiles showing normalized MFI of NLRP3 and TGN signals from (**D**). **F** PCC analysis of NLRP3–TGN38 colocalization (10 cells per group, *n* = 3). Data are presented as mean ± SD. In all quantifications, *n* represents the number of independent experiments. Data shown in (**A**, **C**, **D**) are representative of three independent experiments. Images in (**D**) are quantified in (**F**). Significance determined by two-way ANOVA followed by Tukey’s multiple comparisons test (**B**, **F**); NS not significant.

Previous studies have established that NLRP3 recruitment to the dispersed TGN is mediated by ionic interactions between a conserved polybasic region in NLRP3 and negatively charged PI4P on the TGN membrane [6, 8]. Mutation of four lysine residues (KKKK, amino acids 127–130; referred to as 4KA) in this region abolishes both TGN localization and inflammasome activation, highlighting the motif’s critical role in NLRP3 assembly[6, 8]. Consistent with these reports, the 4KA-mutant mouse NLRP3 expressed in HeLa cells completely lost TGN localization after nigericin stimulation (Fig. 2A). Furthermore, reconstitution with 4KA mouse NLRP3 in *Nlrp3*-deficient BMDMs failed to restore inflammasome activation in response to nigericin or CL097 stimulation (Fig. 2B, C). However, in BMDMs derived from *Nlrp3*^*−*^^/^^*−*^ and *Asc*^*−*^^/^^*−*^ double-knockout mice, the 4KA mutation simultaneously abolished both Golgi localization and phase separation following nigericin stimulation (Fig. 2D–F), confounding efforts to distinguish the individual contributions of these processes to NLRP3 activation.

### Lipid-binding motif deletion abolishes NLRP3 Golgi localization without impairing LLPS and inflammasome activity

**Fig. 3:**
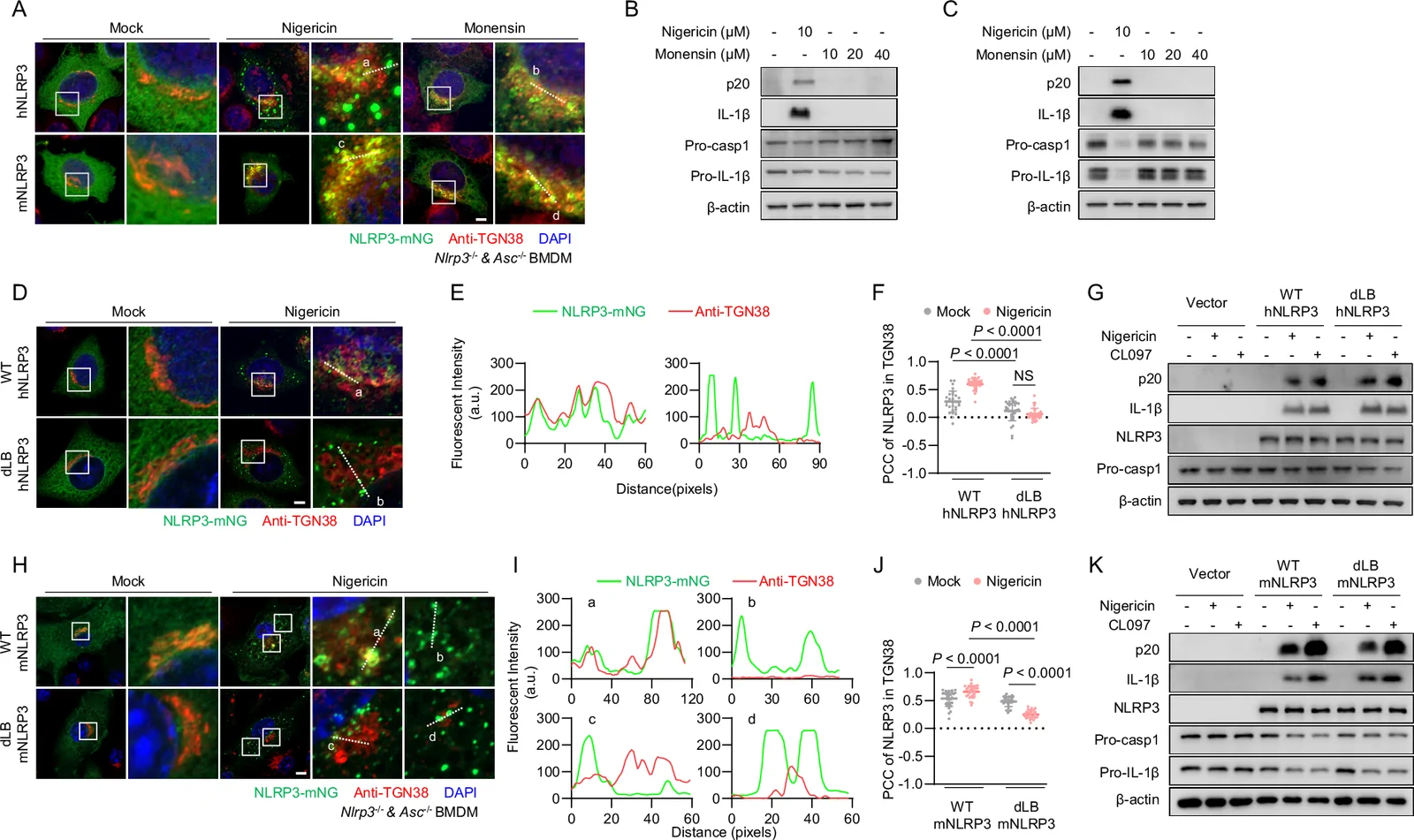
The lipid-binding motif is essential for NLRP3 Golgi localization but not for inflammasome activity. **A** Representative confocal images showing colocalization of the TGN and NLRP3 in HeLa cells stably expressing wild-type human or mouse NLRP3 before and after stimulation with 10 μM nigericin or 10 μM monensin for 60 min. Scale bar, 5 μm. **B** THP-1 cells treated with nigericin or monensin for 60 min. Western blot analysis of cleaved caspase-1 (p20) and IL-1β in supernatants, and NLRP3, pro–caspase-1, pro–IL-1β, and β-actin in cell lysates. **C** BMDMs treated with nigericin or monensin for 60 min. Western blot analysis of cleaved caspase-1 (p20) and IL-1β in supernatants, and NLRP3, pro–caspase-1, pro–IL-1β, and β-actin in cell lysates. **D–F** HeLa cells stably expressing wild-type or dLB mutant human NLRP3 were analyzed for colocalization with the TGN before and after nigericin stimulation. **D** Representative confocal images. Scale bar, 5 μm. **E** Line-scan profiles showing MFI of NLRP3 and TGN signals from (**D**). **F** PCC analysis of NLRP3–TGN38 colocalization (10 cells per group, *n* = 3). **G** *NLRP3*^*−*^^/^^*−*^ THP-1 cells stably expressing wild-type or dLB mutant human NLRP3 were treated with 10 μM nigericin or 40 μM CL097 for 60 min. Western blot analysis of cleaved caspase-1 (p20) and IL-1β in supernatants, and NLRP3, pro–caspase-1, and β-actin in cell lysates. **H–J** *Nlrp3*^*−*^^/^^*−*^ and *Asc*^*−/−*^ double-knockout BMDMs stably expressing wild-type or dLB mutant mouse NLRP3 were analyzed for colocalization with the TGN before and after nigericin stimulation. **H** Representative confocal images. Scale bar, 5 μm. **I** Line-scan profiles showing normalized MFI of NLRP3 and TGN signals from (**H**). **J** PCC analysis of NLRP3–TGN38 colocalization (10 cells per group, *n* = 3). (**K**) *Nlrp3*^*−*^^/^^*−*^ BMDMs complemented with wild-type or dLB mutant mouse NLRP3 were treated with 10 μM nigericin or 40 μM CL097 for 60 min. Western blot analysis of cleaved caspase-1 (p20) and IL-1β in supernatants (SN), and NLRP3, pro–caspase-1, pro–IL-1β, and β-actin in cell lysates (Input). Data are presented as mean ± SD. In all quantifications, *n* represents the number of independent experiments. Representative images shown in (**A**–**D**, **G**, **H**, **K**) are from two or three independent experiments. Images in (**D**, **H**) are quantified in (**F**, **J**), respectively. Significance determined by two-way ANOVA followed by Tukey’s multiple comparisons test (**F**, **J**); NS not significant.

To disentangle the roles of Golgi localization or phase separation in NLRP3 activation, we used monensin, an ionophore structurally related to nigericin and known to disrupt Golgi trafficking [17]. Treatment with monensin robustly enhanced TGN localization of both mouse and human NLRP3 expressed in HeLa cells (Figs. 3A and S2A–C), with fluorescence intensity comparable to that induced by nigericin stimulation (Fig. S2A). Importantly, we confirmed that both monensin and nigericin specifically disrupted the TGN without affecting the distribution of the cis-Golgi marker GM130, indicating that monensin exerts targeted effects rather than causing broad, indiscriminate Golgi damage (Fig. S2D). However, despite this robust TGN accumulation, monensin failed to trigger NLRP3 phase separation (Figs. 3A and S2B, C). Furthermore, monensin treatment did not induce inflammasome activation in THP-1 cells or primary BMDMs (Figs. 3B, C and S2E). Additionally, canonical NLRP3 inhibitors MCC950 [18] and CY-09 [19] nearly abolished nigericin-induced phase separation while promoting NLRP3 accumulation at the TGN (Fig. S2F, G). Together, these results indicate that NLRP3 association with the TGN is a separable event that can occur independently of inflammasome activation.

To further clarify the role of Golgi localization in NLRP3 activation, computational prediction using DEPICTER2[20, 21] was employed to identify potential lipid-binding regions. This analysis identified a principal lipid-binding sequence (SIEEEWMGLLEYLSRISICKMKK, amino acids 112-134) (Fig. S3A). Based on this, a human NLRP3 mutant lacking this lipid-binding sequence (dLB-hNLRP3) was generated (Fig. S3B). Deletion of this region completely abolished stimulus-induced NLRP3 vesicular enrichment while preserving phase separation capacity (Fig. S3D, E). TGN co-localization was eliminated in the dLB-hNLRP3 mutant (Fig. 3D–F). Importantly, immunoblot analysis confirmed that the functional differences were not due to protein instability, as dLB-hNLRP3-mNG maintained expression levels comparable to wild-type human NLRP3 (Fig. S3F). Reconstitution of dLB-hNLRP3 in NLRP3-deficient THP-1 cells fully restored inflammasome activation upon nigericin or CL097 stimulation (Figs. 3G and S3I), demonstrating that the lipid-binding region is essential for TGN localization but dispensable for human NLRP3 activation.

Since the major lipid-binding region in human NLRP3 is not restricted to the polybasic region, we investigated corresponding regions in mouse NLRP3 using DEPICTER2. Surprisingly, the primary predicted lipid-binding motif largely bypassed the polybasic region, localizing instead to adjacent upstream and downstream residues (Fig. S3A). Accordingly, a deletion mutant (dLB-mNLRP3) lacking five upstream residues (RISIC, amino acids 122–126) was constructed (Fig. S3C). Reconstitution of dLB-mNLRP3-mNeonGreen in BMDMs derived from *Nlrp3*^*−*/−^ and *Asc*^−/−^ double-knockout mice showed markedly reduced TGN co-localization under resting conditions, with complete elimination following nigericin stimulation (Fig. 3H, I). Quantitative analyses confirmed minimal spatial overlap between NLRP3 and TGN markers in both basal and activated states (Fig. 3J). Strikingly, loss of lipid-binding capacity did not impair the formation of NLRP3 phase-separated condensates (Figs. 3H and S3G). Similarly, immunoblot analysis demonstrated that the dLB-mNLRP3-mNG mutant was stably expressed at levels comparable to wild-type mouse NLRP3 in BMDMs (Fig. S3H). Consistent with the human findings, dLB-mNLRP3 fully restored inflammasome activation in response to nigericin or CL097 stimulation in NLRP3-deficient BMDMs (Figs. 3K and S3J).

Thus, these results establish that the lipid-binding region is critical for TGN localization but dispensable for NLRP3 inflammasome activation in both species, indicating that Golgi localization is not essential for NLRP3 inflammasome activation.

### The FISNA domain IDR mediates both NLRP3 Golgi localization and phase separation

**Fig. 4:**
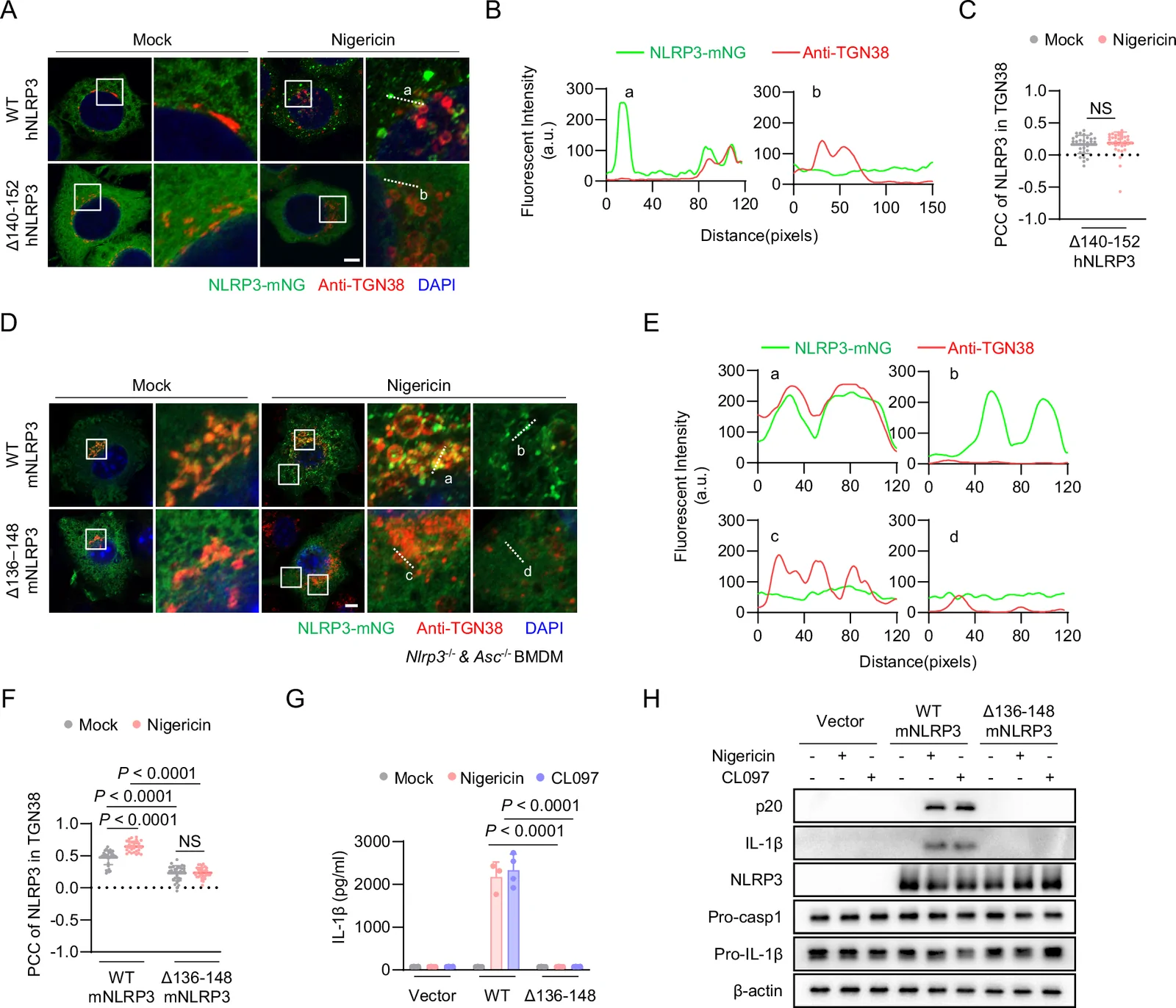
The FISNA domain IDR is essential for both NLRP3 Golgi localization and phase separation. **A**–**C** HeLa cells stably expressing wild-type or Δ140-152 mutant human NLRP3 were analyzed for colocalization with the TGN before and after nigericin stimulation. **A** Representative confocal images. Scale bar, 5 μm. **B** Line-scan profiles showing MFI of NLRP3 and TGN signals from (**A**). **C** PCC analysis of Δ140-152 NLRP3–TGN38 colocalization (10 cells per group, *n* = 3). **D**–**F** *Nlrp3*^*−*/−^ and *Asc*^*−*^^/−^ double-knockout BMDMs stably expressing wild-type or Δ136–148 mutant mouse NLRP3 were analyzed for colocalization with the TGN before and after nigericin stimulation. **D** Representative confocal images. Scale bar, 5 μm. **E** Line-scan profiles showing normalized MFI of NLRP3 and TGN signals from (**D**). **F** PCC analysis of NLRP3–TGN38 colocalization (10 cells per group, *n* = 3). **G**, **H** *Nlrp3*^*−*/−^ BMDMs complemented with wild-type or Δ136–148 mutant mouse NLRP3 were stimulated with 10 μM nigericin or 40 μM CL097 for 60 min. **G** IL-1β levels in supernatants measured by ELISA (*n* = 3). **H** Western blot analysis of cleaved caspase-1 (p20) and IL-1β in supernatants, and NLRP3, pro–caspase-1, pro–IL-1β, and β-actin in cell lysates. Data are presented as mean ± SD. In all quantifications, *n* represents the number of independent experiments. Data shown in (**A**, **D**, **H**) are representative of two or three independent experiments. Images in (**A**, **D**) are quantified in (**C**, **F**), respectively. Significance determined by unpaired two-tailed Student’s t-test (**C**) or two-way ANOVA followed by Tukey’s multiple comparisons test (**F**, **G**); NS not significant.

A recent study showed that NLRP3 undergoes palmitoylation-mediated LLPS through an IDR within its FISNA domain [11]. To validate this, the IDR segment (amino acids 140-152, termed Δ140-152 hNLRP3) was deleted in human NLRP3 expressed in HeLa cells. This mutant confirmed the reported suppression of nigericin-induced NLRP3 phase separation (Figs. 4A and S4A, B). Moreover, it caused complete loss of NLRP3 co-localization with the TGN (Figs. 4A–C and S4A, B). To assess evolutionary conservation, a corresponding mouse NLRP3 mutant lacking residues 136–148 (termed Δ136–148 mNLRP3) was generated and stably expressed in BMDMs from *Nlrp3*^*−/−*^ and *Asc*^*−*^^/^^*−*^ double-knockout mice. Consistent with the human results, Δ136–148 mNLRP3 disrupted both TGN localization and phase separation capacity of NLRP3 (Fig. 4D–F). Furthermore, reconstitution with Δ136–148 mNLRP3 in *Nlrp3*^*−*^^/^^*−*^ BMDMs failed to restore inflammasome activation upon stimulation (Fig. 4G–H).

Collectively, these results establish that the IDR within the FISNA domain coordinately regulates both TGN localization and phase separation, precluding a clear distinction between the roles of LLPS and Golgi localization in NLRP3 activation.

### The C-terminal IDR is required for phase separation and NLRP3 inflammasome activity but not Golgi localization

**Fig. 5:**
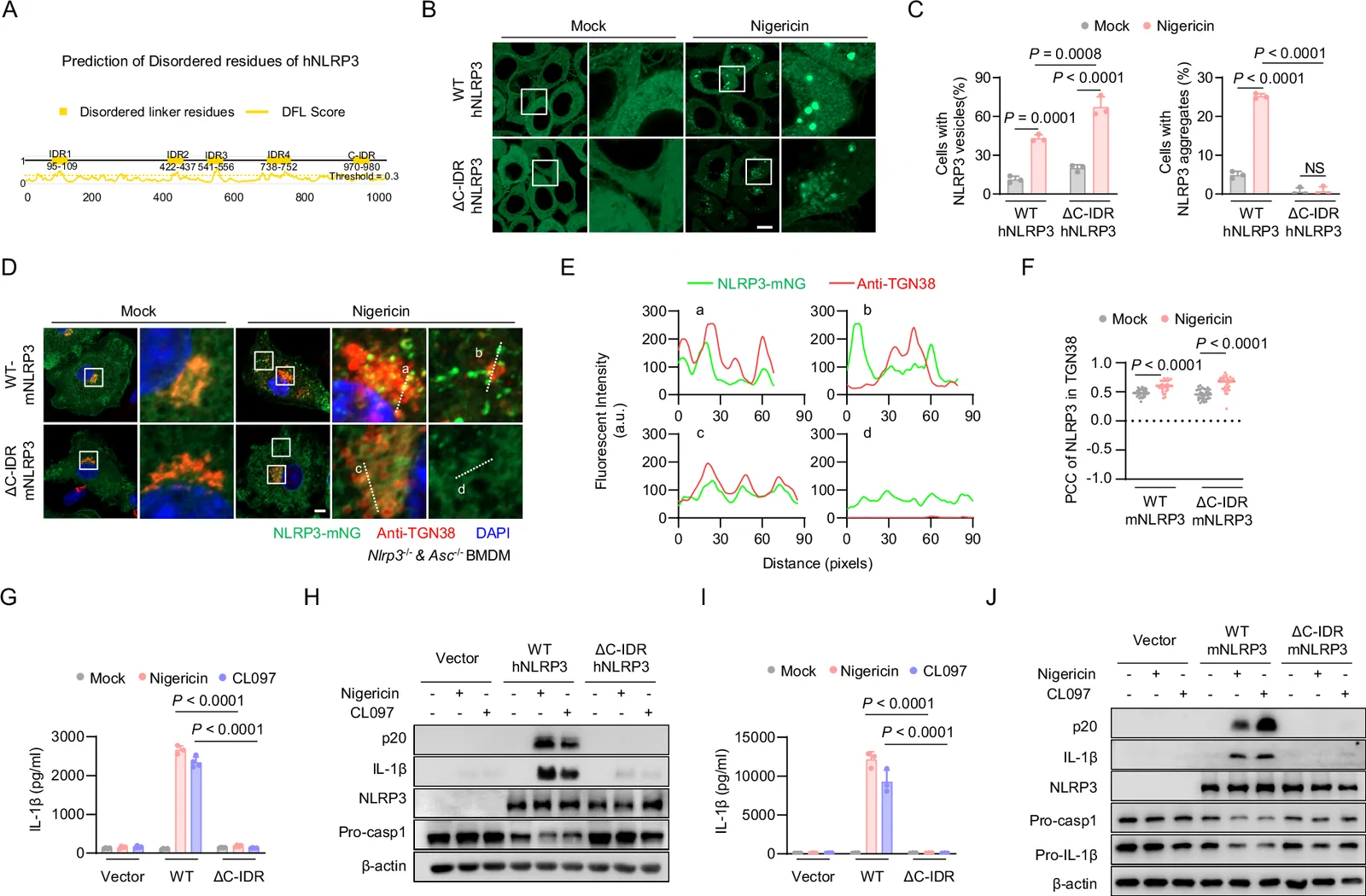
The C-terminal IDR is required for NLRP3 inflammasome activity but not Golgi localization. **A** Plot showing shorter intrinsic disorder prediction of human NLRP3 analyzed by DEPICTER2. Five regions exceeding 10 consecutive disordered residues were identified as potential IDRs. **B** Representative images of HeLa cells stably expressing wild-type or ΔC-IDR mutant human NLRP3 before and after stimulation with 10 μM nigericin for 60 min. Scale bar, 10 μm. **C** Quantification of cells containing NLRP3 vesicles or aggregates (>60 cells per group, *n* = 3). (**D–F**) *Nlrp3*^*−*/^^*−*^ and *Asc*^*−*^^/−^ double-knockout BMDMs stably expressing wild-type or ΔC-IDR mutant mouse NLRP3 were analyzed for colocalization with the TGN before and after nigericin stimulation. **D** Representative confocal images. Scale bar, 5 μm. **E** Line-scan profiles showing normalized MFI of NLRP3 and TGN signals from (**D**). (**F**) PCC analysis of NLRP3–TGN38 colocalization (10 cells per group, *n* = 3). **G**, **H** *NLRP3*^*−*^^/−^ THP-1 cells stably expressing wild-type or ΔC-IDR mutant human NLRP3 were stimulated with 10 μM nigericin or 40 μM CL097 for 60 min. **G** IL-1β levels in supernatants measured by ELISA (*n* = 3). **H** Western blot analysis of cleaved caspase-1 (p20) and IL-1β in supernatants, and NLRP3, pro–caspase-1, pro–IL-1β, and β-actin in cell lysates. **I**, **J** *Nlrp3*^*−*^^/−^ BMDMs complemented with wild-type or ΔC-IDR mutant mouse NLRP3 were stimulated with 10 μM nigericin or 40 μM CL097 for 60 min. **I** IL-1β levels in supernatants measured by ELISA (*n* = 3). **J** Western blot analysis of cleaved caspase-1 (p20) and IL-1β in supernatants, and NLRP3, pro–caspase-1, pro–IL-1β, and β-actin in cell lysates. Data are presented as mean ± SD. In all quantifications, *n* represents the number of independent experiments. Data shown in (**B**, **D**, **H**, **J**) are representative of two or three independent experiments. Images in (**B**, **D**) are quantified in (**C**), (**F**), respectively. Significance determined by two-way ANOVA followed by Tukey’s multiple comparisons test (**C**, **F**, **G**, **I**); NS not significant.

To identify regions essential for NLRP3 phase separation, we examined other IDRs, known drivers of LLPS [22]. Analysis with the flDPnn algorithm on the DEPICTER2 platform [20, 21] predicted a single IDR in human NLRP3 (residues 130–141), overlapping with the previously characterized lipid-binding domain (residues 112–134). To detect shorter disordered segments, DFLpred analysis was applied [23], revealing five additional IDRs exceeding ten amino acids each (Fig. 5A).

To evaluate their functional relevance, deletion mutants corresponding to each predicted IDR were constructed and stably expressed in HeLa cells. Deletion of ΔIDR1-hNLRP3 (residues 95–109) showed no detectable effect on NLRP3 phase separation (Fig. S4C). In contrast, deletions of ΔIDR2-hNLRP3 (residues 422–437), ΔIDR3-hNLRP3 (residues 541–556), and ΔIDR4-hNLRP3 (residues 738–752) impaired both NLRP3 phase separation and TGN localization (Fig. S4C). Notably, deletion of the C-terminal IDR motif SLRKLSLGNND (residues 970–980) in ΔC-IDR-hNLRP3 specifically abolished condensate formation while preserving TGN localization (Fig. 5B, C). This suppression of phase separation was consistently observed following CL097 stimulation or K^+^-free medium treatment (Fig. S4E), and was replicated in *NLRP3*^*−/−*^ and *ASC*^*−*^^/^^*−*^ double-knockout THP-1 cells (Fig. S4D).

To assess the evolutionary conservation, a corresponding mouse NLRP3 mutant lacking residues 967–977 (ΔC-IDR-mNLRP3) was generated and stably expressed in BMDMs derived from *Nlrp3*^*−*^^/^^*−*^ and *Asc*^*−*^^/^^*−*^ double-knockout mice. Consistent with the human mutant, ΔC-IDR-mNLRP3 abolished phase-separated condensate formation without affecting TGN co-localization (Figs. 5D–F and S4F). Immunoblotting confirmed that reconstituted WT and ΔC-IDR mutants were expressed at endogenous levels in both cell types (Fig. S5A; B), ruling out protein instability. These findings establish an evolutionarily conserved role for this C-terminal IDR in promoting biomolecular condensation independently of TGN localization.

We then examined the role of this C-terminal IDR in inflammasome activation. Reconstitution of ΔC-IDR-hNLRP3 in NLRP3-deficient THP-1 cells failed to restore inflammasome activation after nigericin or CL097 stimulation (Fig. 5G, H). Similarly, complementation with ΔC-IDR-mNLRP3 in *Nlrp3*-deficient BMDMs did not rescue inflammasome activation (Fig. 5I, J). Further finer mapping confirmed that the basic R972-K973 motif is essential for condensate formation (Fig. S5C). To determine whether phase separation serves as a universal prerequisite across distinct activation modalities, we examined the human-specific alternative inflammasome pathway [24, 25]. We found that condensate formation and subsequent IL-1β secretion were strictly dependent on the C-terminal IDR during this alternative activation (Fig. S5D, E). Assessment of downstream ASC speck formation revealed that the dLB mutant robustly recruited ASC, while the ΔC-IDR mutant failed to assemble these complexes (Fig. S5F, G). Together, these consolidated data demonstrate that the C-terminal IDR is essential for functional NLRP3 inflammasome assembly despite preserved membrane localization, firmly establishing that biomolecular condensation, rather than TGN localization, is the universal and absolute prerequisite for downstream signaling.

## Discussion

The precise molecular events bridging NLRP3 sensing to inflammasome assembly have long been a subject of debate [3, 6, 8, 11, 15, 26–30]. While initial models emphasized the necessity of NLRP3 translocation to the TGN via ionic interactions with PI4P [6, 8], emerging evidence has pointed towards LLPS-driven biomolecular condensation as a dominant mechanism for nucleating the supramolecular complex [11]. Resolving this dichotomy has been hindered by the fact that previously characterized motifs—specifically the polybasic region and the FISNA domain IDR—appear to govern both processes simultaneously. In this study, we successfully disentangled these two phenomena. By identifying and characterizing distinct molecular determinants for membrane binding and condensate formation, we provide compelling evidence that phase separation is the intrinsic driver of NLRP3 activation, whereas Golgi localization is a separable and dispensable event.

Our findings challenge the prevailing dogma that TGN recruitment is an absolute prerequisite for NLRP3 activation [6, 8]. By utilizing DEPICTER2 [20] algorithms to map lipid-binding sequences, we generated deletion mutants (dLB-NLRP3) in both human and mouse orthologs that completely fail to localize to the TGN. Crucially, these mutants retained the ability to undergo phase separation and fully restored inflammasome activity in reconstitution assays. This result fundamentally shifts our understanding of the spatial requirements for the inflammasome, aligning with recent observations that certain activating mutants function independently of the TGN [9, 10].

Furthermore, our pharmacological data using monensin reinforces this conclusion from the perspective of sufficiency. Monensin treatment robustly drove NLRP3 to the TGN yet failed to induce phase separation or inflammasome activation [15]. This creates a clear distinction: while TGN association often correlates with activation under physiological conditions (likely due to the overlapping function of the polybasic region), it is neither necessary nor sufficient for the assembly of a functional inflammasome. In this context, we interpret TGN localization as a stimulus-specific event rather than a universal requirement for NLRP3 activation. The previously observed “necessity” of the Golgi was likely an artifact of mutations (such as the 4KA mutant) that inadvertently disrupted the protein’s phase-separating propensity alongside its lipid-binding capacity [6, 8].

In contrast to Golgi localization, our data establish LLPS as the essential biophysical mechanism underlying NLRP3 activation. We identified a previously uncharacterized evolutionary conserved C-terminal IDR (residues 970–980 in humans) that is specific to the phase separation process[20, 23]. Deletion of this motif (ΔC-IDR) spared Golgi localization but abolished condensate formation and subsequent inflammasome activity.

This finding is significant for two reasons. First, it confirms that membrane accumulation of NLRP3 alone—even at the high densities provided by TGN sorting—is inadequate to trigger caspase-1 activation without the biophysical transition into a condensate state. Second, it highlights the multivalent nature of NLRP3 regulation. While the N-terminal FISNA IDR participates in both membrane binding and oligomerization [11], the C-terminal IDR appears to function as a specialized region dedicated to driving LLPS required for stable inflammasome assembly. The dynamic properties observed in our FRAP and live-cell imaging experiments further validate that these aggregates are bona fide liquid droplets, capable of fusion and internal rearrangement, rather than non-specific protein aggregates.

A technical but critical insight from our study resolves discrepancies regarding mouse versus human NLRP3 behavior. We observed that heterologous expression of murine NLRP3 in human HeLa cells fails to recapitulate the phase separation seen in native contexts. However, when expressed in primary murine macrophages, mouse NLRP3 exhibits clear droplet formation similar to its human counterpart. This suggests that the cellular microenvironment—potentially involving species-specific chaperones or post-translational modifiers—plays a crucial role in licensing NLRP3 for phase separation. Future studies utilizing murine NLRP3 should prioritize homologous expression systems to avoid overlooking this conserved biophysical behavior.

The identification of phase separation as the primary activation mechanism, independent of Golgi trafficking, opens new avenues for therapeutic intervention. Current inhibitors like MCC950 [18] are known to affect NLRP3 conformation, and our data show they also block phase separation while trapping NLRP3 at the Golgi. However, targeting the lipid-binding interface may prove ineffective, as our dLB mutants demonstrate that activation can bypass the Golgi entirely. Instead, precision therapies aimed specifically at disrupting the interactions mediated by the C-terminal IDR could provide a potent strategy to suppress sterile inflammation in diseases such as gout[31], Alzheimer’s[32], and type 2 diabetes[33], without disrupting global Golgi function or trafficking pathways.

Limitations of this study include reliance on overexpression systems, which may amplify artifactual behaviors, though mitigated by knockout-reconstitution in primary cells. Future investigations should probe endogenous NLRP3 dynamics using CRISPR-edited models and explore how upstream stressors (e.g., mitochondrial dysfunction, K^+^ efflux, or others) interface with LLPS [22]. While WT condensates exhibit liquid-like properties, quantitatively comparing FRAP kinetics across different mutants remains for future studies. Additionally, structural studies of the C-terminal IDR in condensates could unveil atomic-level drivers of phase behavior. Finally, although NLRP3 LLPS was robustly induced in multiple cellular contexts (HeLa cells, THP-1 cells, and primary BMDMs), attempts to reconstitute phase separation in vitro using purified NLRP3 were unsuccessful, indicating that essential cofactors or cellular cues are likely required.

In summary, we have redefined the regulatory landscape of the NLRP3 inflammasome. By genetically and functionally uncoupling spatial localization from biophysical condensation, we conclude that while NLRP3 often visits the Golgi, it is the phase separation driven by specific intrinsic disordered regions that ultimately licenses the immune response.

## Materials and methods

### Mice

C57BL/6J mice were obtained from the Shanghai SLAC Laboratory Animal Ltd Corp. (Shanghai, China). *Nlrp3* ^−^^/−^ mice were kindly provided by Dr. Jurg Tschopp’s group. *Asc*^−/−^ mice were provided by the laboratory of Dr. Vishva M. Dixit. All animals were maintained in a specific pathogen-free facility under a 12-h light/dark cycle at 22–24 °C and 40–70% humidity, with unrestricted access to food and water. All mouse experiments were approved by the Ethics Committee of the University of Science and Technology of China (USTC) (Approval No. USTCACUC29080123072). The study is compliant with all relevant ethical regulations regarding animal research.

### Antibodies and Reagents

Nigericin, CL097, MCC950, and CY-09 were obtained from Selleck. Lipopolysaccharide (LPS; L2630) and phorbol 12-myristate 13-acetate (PMA; P1585) were purchased from Sigma. K^+^-free medium was prepared as described previously[34], containing 135 mM NaCl, 1 mM MgCl₂, 1 mM CaCl₂, 20 mM HEPES (pH 7.4), and 10 mM glucose. Anti-mouse caspase-1 (p20) (AG-20B-0048-C100) and anti-NLRP3 (AG-20B-0014) antibodies were obtained from Adipogen. Anti-human Cleaved-IL-1β (D3A3Z), anti-mouse ASC (D2W8U), and anti-mouse TGN38 (E2T4P) antibodies were obtained from Cell Signaling Technology. Anti-human TGN38 (13573-1-AP) and anti-β-actin (66009-1-Ig) antibodies were obtained from Proteintech.

### Cell culture and stimulation

HEK293T, HeLa, and THP-1 cells were obtained from the American Type Culture Collection (ATCC). Bone marrow–derived macrophages (BMDMs) were isolated and cultured as previously described[35]. BMDMs, HEK293T, and HeLa cells were maintained in Dulbecco’s modified Eagle’s medium (DMEM; Gibco) supplemented with 10% fetal bovine serum (FBS; Gibco), while THP-1 cells were cultured in RPMI 1640 medium (Gibco) containing 10% FBS. All cells were incubated at 37 °C in a humidified atmosphere with 5% CO₂ and routinely tested to confirm the absence of mycoplasma contamination.

For NLRP3 inflammasome activation, THP-1 cells were seeded in 24-well plates and treated with phorbol 12-myristate 13-acetate (PMA, 100 nM) for 3 h to promote adherence. The medium was then replaced, and cells were allowed to differentiate for 48 h. Differentiated THP-1 cells were subsequently stimulated with nigericin (10 μM) for 50 min, CL097 (40 μM) for 1 h, or subjected to potassium-free (K^+^-free) conditions for 1 h.

### Plasmids and stable cell lines

Lentiviral vectors (pLVX) for protein expression were provided by Dr. Shu Zhu (University of Science and Technology of China). Human NLRP3 coding sequence (CDS) was obtained via reverse transcription of RNA extracted from THP-1 cells, and mouse NLRP3 CDS was obtained from BMDM RNA. Wild-type and mutant NLRP3 constructs were cloned into pLVX vectors and fused at the C terminus to mNeonGreen via a GS-linker.

Lentiviral particles were produced by co-transfecting HEK293T cells with pLVX constructs and packaging plasmids (Δ8.9 and VSVG) at a ratio of 6:3:2. Medium was replaced 12 h post-transfection, and viral supernatants were collected 36–48 h later, followed by centrifugation at 6000 rpm for 5 min. Viral supernatants were added to target cell cultures, with medium replacement after 12–18 h. Selection or downstream experiments were performed 24 h post-infection.

Gene silencing was achieved using a CRISPR–Cas9 system with the lentiCRISPR v2 plasmid (Addgene no. 52961). Lentiviral production was performed as described above. The following sgRNAs were cloned downstream of the U6 promoter: Human *NLRP3*: GTACTTCTTACGGTAATCTA; Human *PYCARD*: ACCGGGCTGCGCTTATCGCG.

### Immunostaining and fluorescence microscopy

For immunostaining, cells were fixed with pre-cooled methanol at 4 °C for 5 min, followed by permeabilization with 0.01% Triton X-100 in PBS. After washing, cells were incubated with primary antibodies overnight at 4 °C, followed by incubation with appropriate secondary antibodies at room temperature for 1 h. Coverslips were then mounted and imaged using a Zeiss LSM 980 Airyscan confocal microscope. For live-cell imaging, cells were seeded onto 24-well glass-bottom plates and imaged using the same microscope under live-imaging conditions. For droplet fusion assays, cells were observed using the Zeiss LSM 980 microscope, and images were acquired every 10 s. For fluorescence recovery after photobleaching (FRAP) experiments, photobleaching was performed on three regions of interest (ROIs) using a 488 nm laser at 80–100% power. Post-bleaching images were collected every 10 s using the Zeiss LSM 980 Airyscan microscope.

### NLRP3 reconstitution in BMDM

Mouse NLRP3–mNeonGreen (mNG) expression constructs were generated using the MSCV vector, following the same cloning strategy described above for the pLVX system. Retroviral packaging was performed using the pCL-Eco plasmid at a plasmid ratio of 6:5. Viral supernatants were harvested using the same procedure as for lentivirus production. BMDMs were seeded onto 24-well glass-bottom plates and cultured overnight. Retroviral supernatant was added to the medium the following day, and the medium was replaced after 12–18 h of incubation. Two days after transduction, BMDMs were primed with 50 ng/mL LPS for 3 h, stimulated with nigericin for 30 min, and imaged using an LSM980 Airyscan microscope.

### Immunoblotting and ELISA

Cell lysates were denatured in sample buffer at 100 °C for 10 min, and equal amounts of protein were resolved by SDS-PAGE at 88 V for 2 h. Proteins were transferred onto PVDF membranes at 88 V for 1 h. Membranes were blocked with 5% nonfat milk for 10 min at room temperature, followed by overnight incubation at 4 °C with primary antibodies diluted in 5% BSA in PBS. After three washes with PBST (5 min each), membranes were incubated with appropriate secondary antibodies in 5% nonfat milk for 1 h at room temperature. Following three additional PBST washes, signals were detected by chemiluminescence. For ELISA, Cell culture supernatants were analyzed for human IL-1β (BD, 557953), mouse IL-1β (R&D) according to the manufacturer’s instructions.

### Image analysis

Image processing and quantitative analyses were performed in Fiji. Colocalization was evaluated using the Pearson correlation coefficient. Quantification of NLRP3–TGN38 association was carried out by manually delineating the TGN38-positive region to define the corresponding region of interest (ROI). Linear fluorescence-intensity measurements were obtained by drawing a line with the Straight tool at the designated position, followed by extraction of intensity profiles using the Plot Profile function. For all experiments, images were acquired randomly under identical microscope settings (laser power, gain), and pixel intensities were kept below saturation.

### Statistical analyses

All data are presented as mean ± SD. Statistical analyses were performed using GraphPad Prism 8. For experiments with small sample sizes, data distribution was assumed to be normal based on historical data of similar experimental setups, but this was not formally tested. Comparisons between two groups were assessed using unpaired two-tailed Student’s t-tests, whereas multiple-group comparisons were analyzed using one-way or two-way ANOVA, as appropriate. No data were excluded from the analyses. Sample sizes were selected on the basis of preliminary results to ensure adequate power. The exact values of *n*, statistical significance and number of replicates are reported.

### Use of large language models

Large language model (LLM) assistance (Grok) was used solely for language polishing, grammar correction, and improving the clarity and readability of the manuscript. No scientific content, data interpretation, or analytical conclusions were generated by the LLM. All edits made with LLM assistance were manually reviewed and verified by the authors.

## Supplementary information

Supplemental Material

Original Western Blots

Nigericin-Induced NLRP3 Droplet Formation (Related to Fig. 1J)

CL097-Induced NLRP3 Droplet Formation (Related to Fig. S1F)

K+ free Medium–Induced NLRP3 Droplet Formation (Related to Fig. S1I)

Supplementary figure1

Supplementary figure2

Supplementary figure3

Supplementary figure4

Supplementary figure5

## Data Availability

The data supporting the findings of this study are available from the corresponding authors upon reasonable request.
