# Supplementary material for: Phase separation drives NLRP3 inflammasome activation independently of Golgi localization
Source: Cell Death Dis. 2026 Jul 6;17(1):839. doi: 10.1038/s41419-026-09059-4 (PMC13616974; doi:10.1038/s41419-026-09059-4)
Supplement: Supplementary file 1 [file 41419_2026_9059_MOESM10_ESM.pdf]

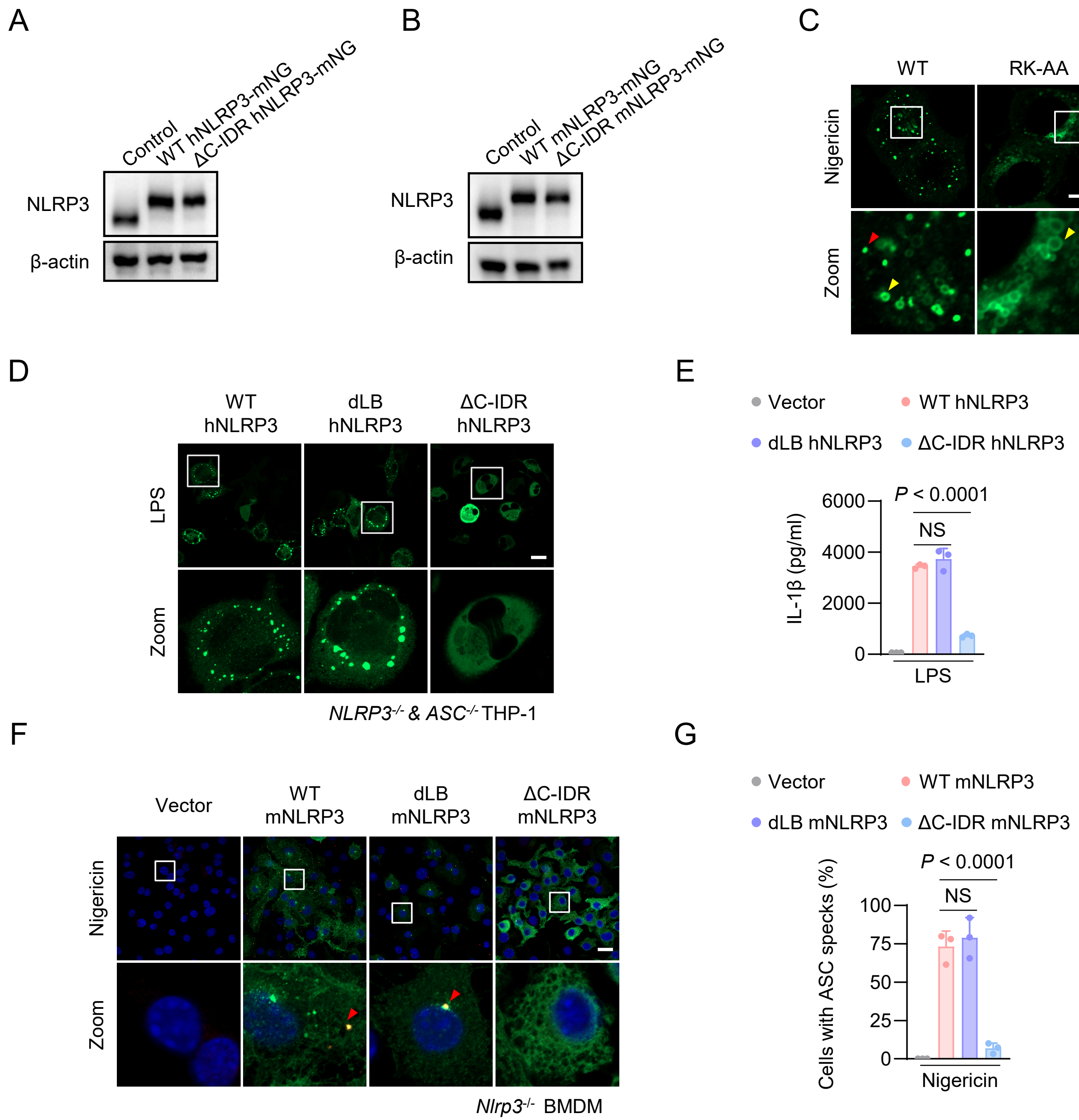
A
Control
WT hNLRP3-mNG
ΔC-IDR hNLRP3-mNG
NLRP3
β-actin
B
Control
WT mNLRP3-mNG
ΔC-IDR mNLRP3-mNG
NLRP3
β-actin
C
WT
RK-AA
Nigericin
Zoom
D
WT hNLRP3
dLB hNLRP3
ΔC-IDR hNLRP3
LPS
Zoom
NLRP3-/- & ASC-/- THP-1
E
Vector
WT hNLRP3
dLB hNLRP3
ΔC-IDR hNLRP3
P < 0.0001
NS
IL-1β (pg/ml)
6000
4000
2000
0
LPS
F
Vector
WT mNLRP3
dLB mNLRP3
ΔC-IDR mNLRP3
Nigericin
Zoom
Nlrp3-/- BMDM
G
Vector
WT mNLRP3
dLB mNLRP3
ΔC-IDR mNLRP3
P < 0.0001
NS
Cells with ASC specks (%)
100
75
50
25
0
Nigericin
