# Supplementary material for: Phase separation drives NLRP3 inflammasome activation independently of Golgi localization
Source: Cell Death Dis. 2026 Jul 6;17(1):839. doi: 10.1038/s41419-026-09059-4 (PMC13616974; doi:10.1038/s41419-026-09059-4)

Fig. 2C

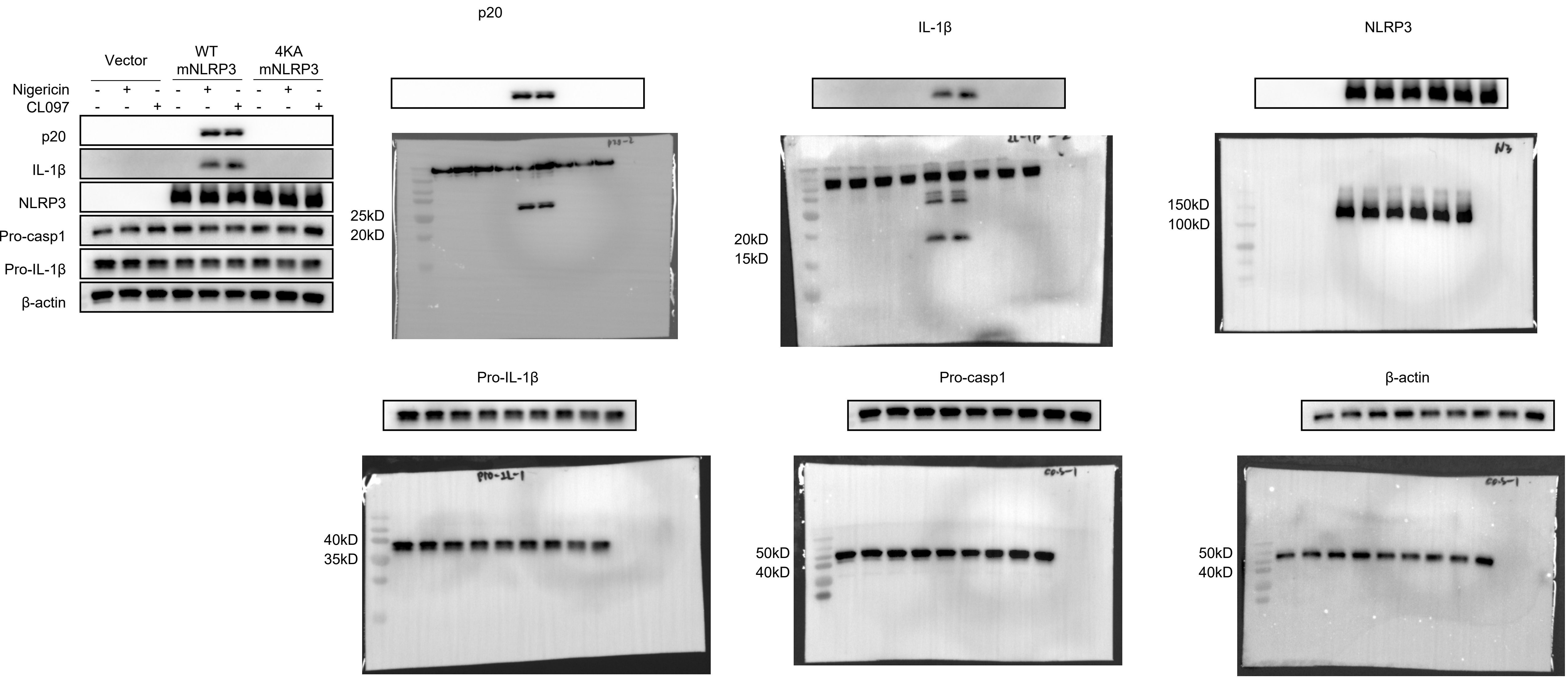

Vector
WT mNLRP3
4KA mNLRP3
Nigericin - + - - + - - + -
CL097 - - + - - + - - +
p20
IL-1β
NLRP3
Pro-casp1
Pro-IL-1β
β-actin
p20
25kD
20kD
IL-1β
20kD
15kD
NLRP3
150kD
100kD
Pro-IL-1β
40kD
35kD
Pro-casp1
50kD
40kD
β-actin
50kD
40kD

Fig. 3B

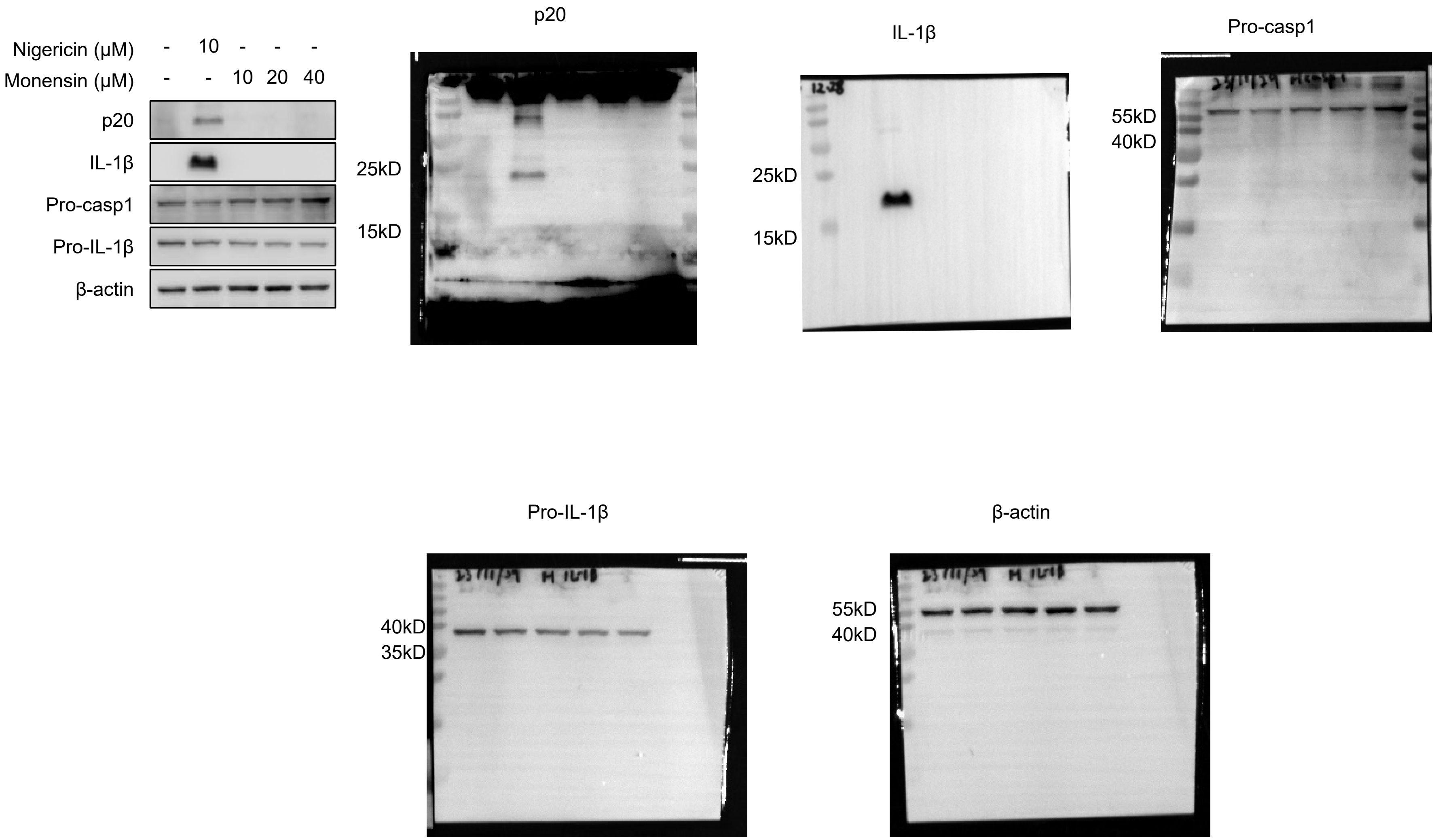

Nigericin (μM) - 10 - - -
Monensin (μM) - - 10 20 40
p20
IL-1β
Pro-casp1
Pro-IL-1β
β-actin
p20
25kD
15kD
IL-1β
25kD
15kD
Pro-casp1
55kD
40kD
Pro-IL-1β
40kD
35kD
β-actin
55kD
40kD

Fig. 3C

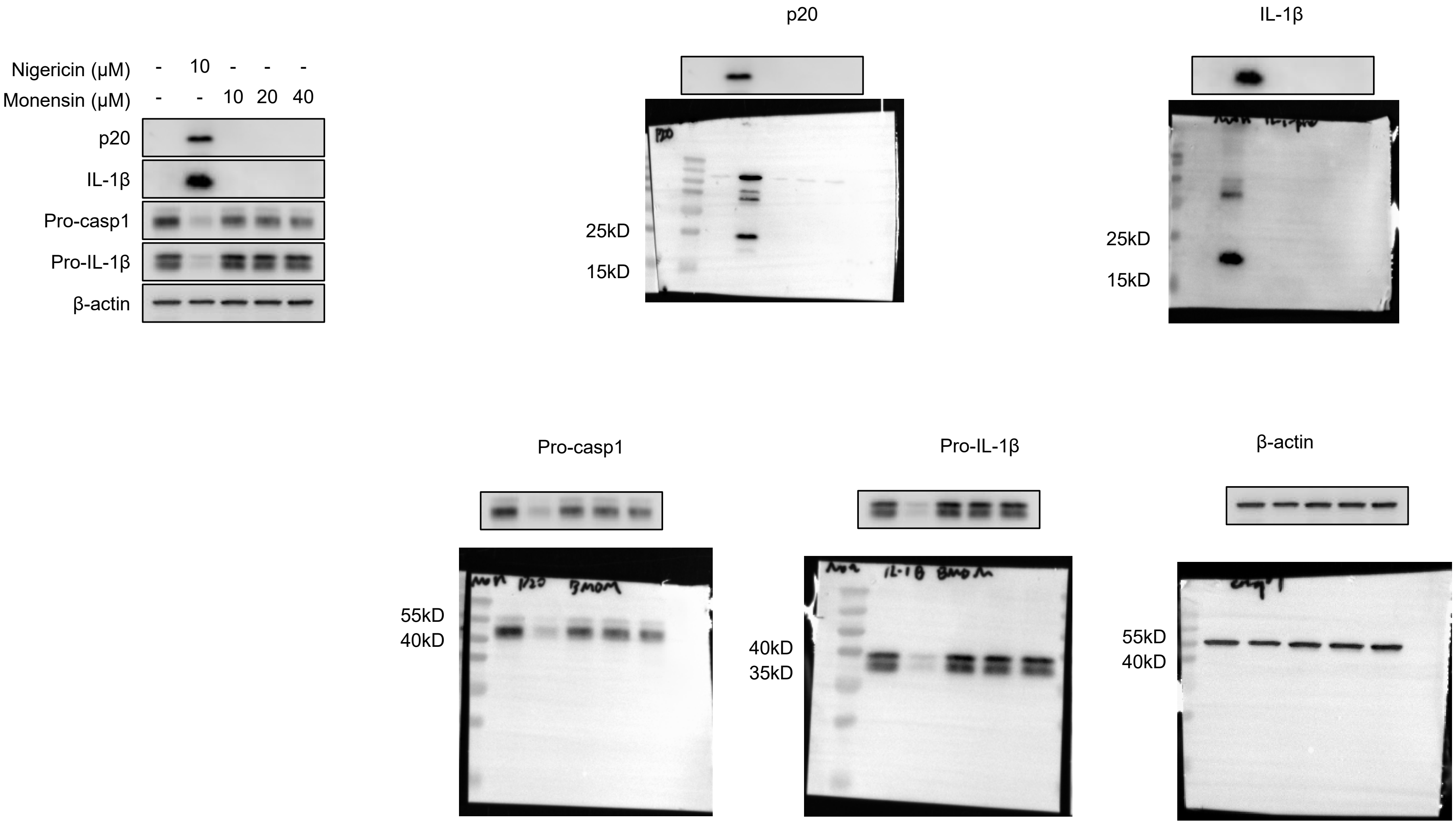
Nigericin (μM) - 10 - - -
Monensin (μM) - - 10 20 40
p20
IL-1β
Pro-casp1
Pro-IL-1β
β-actin
p20
25kD
15kD
IL-1β
25kD
15kD
Pro-casp1
55kD
40kD
Pro-IL-1β
40kD
35kD
β-actin
55kD
40kD

Fig. 3G

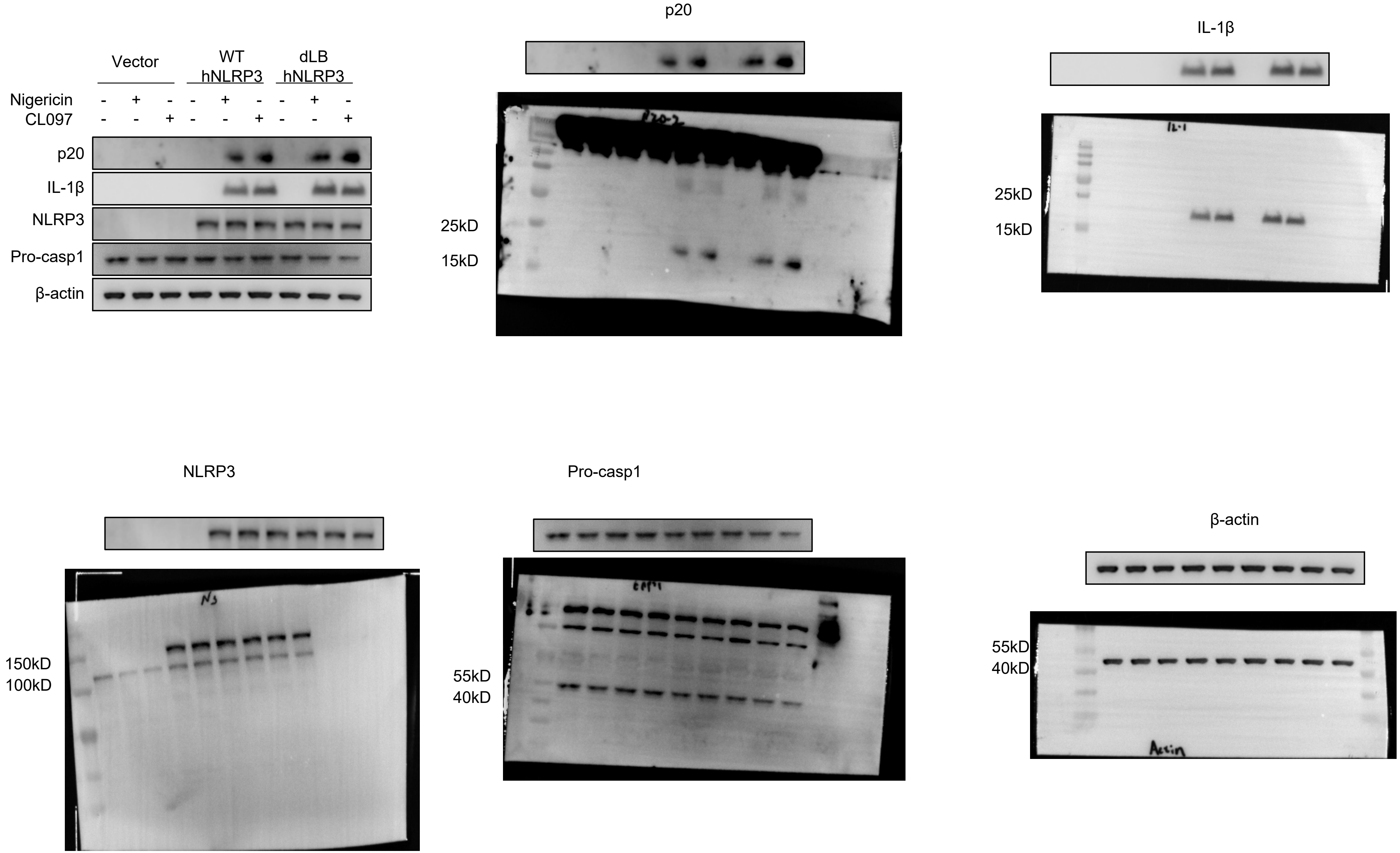
Vector
WT hNLRP3
dLB hNLRP3
Nigericin - + - - + - - + -
CL097 - - + - - + - - +
p20
IL-1β
NLRP3
Pro-casp1
β-actin
p20
25kD
15kD
IL-1β
25kD
15kD
NLRP3
150kD
100kD
Pro-casp1
55kD
40kD
β-actin
55kD
40kD

Fig. 3K

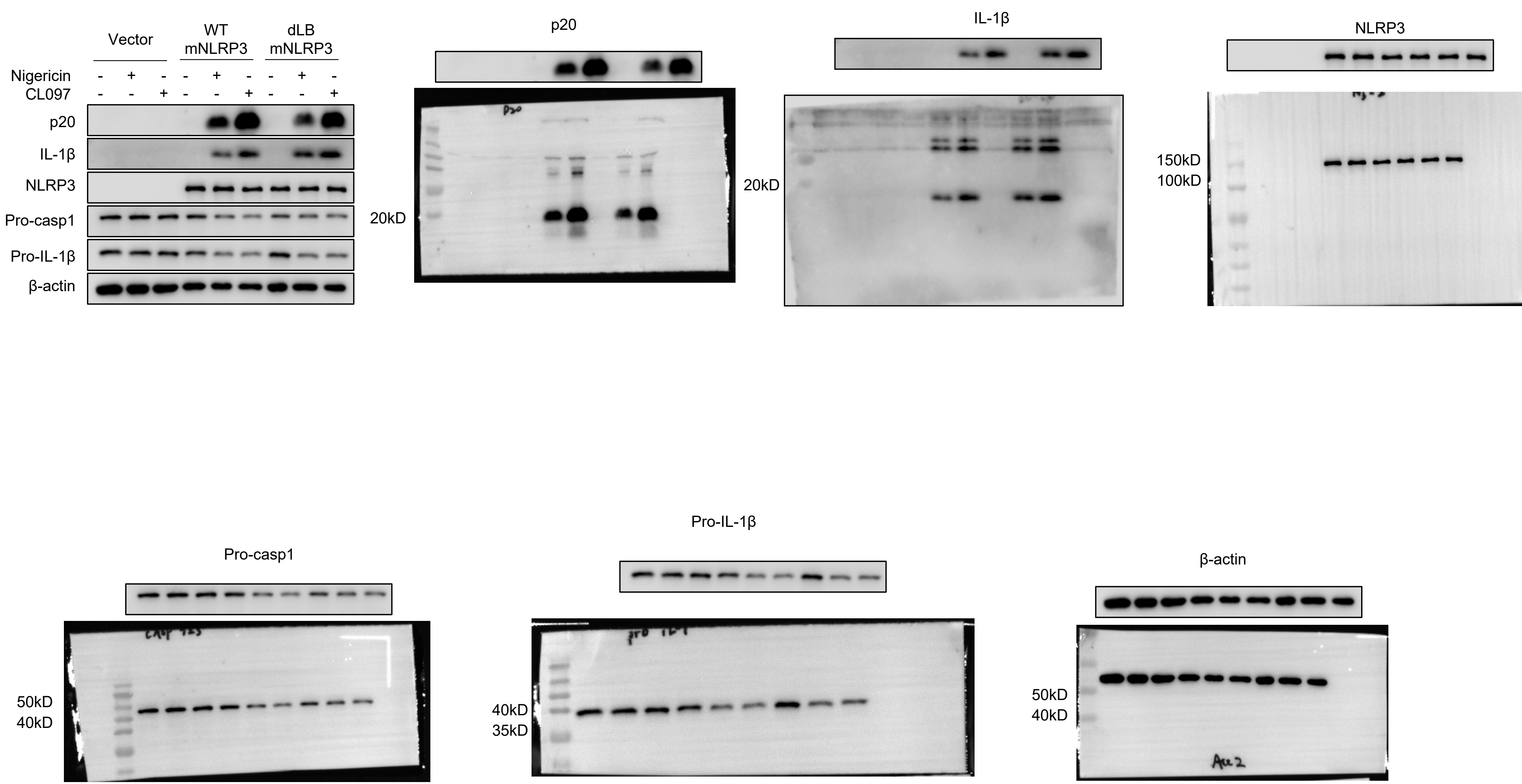
Vector
WT mNLRP3
dLB mNLRP3
Nigericin - + - - + - - + -
CL097 - - + - - + - - +
p20
IL-1β
NLRP3
Pro-casp1
Pro-IL-1β
β-actin
p20
20kD
IL-1β
20kD
NLRP3
150kD
100kD
Pro-casp1
50kD
40kD
Pro-IL-1β
40kD
35kD
β-actin
50kD
40kD

Fig. 4H

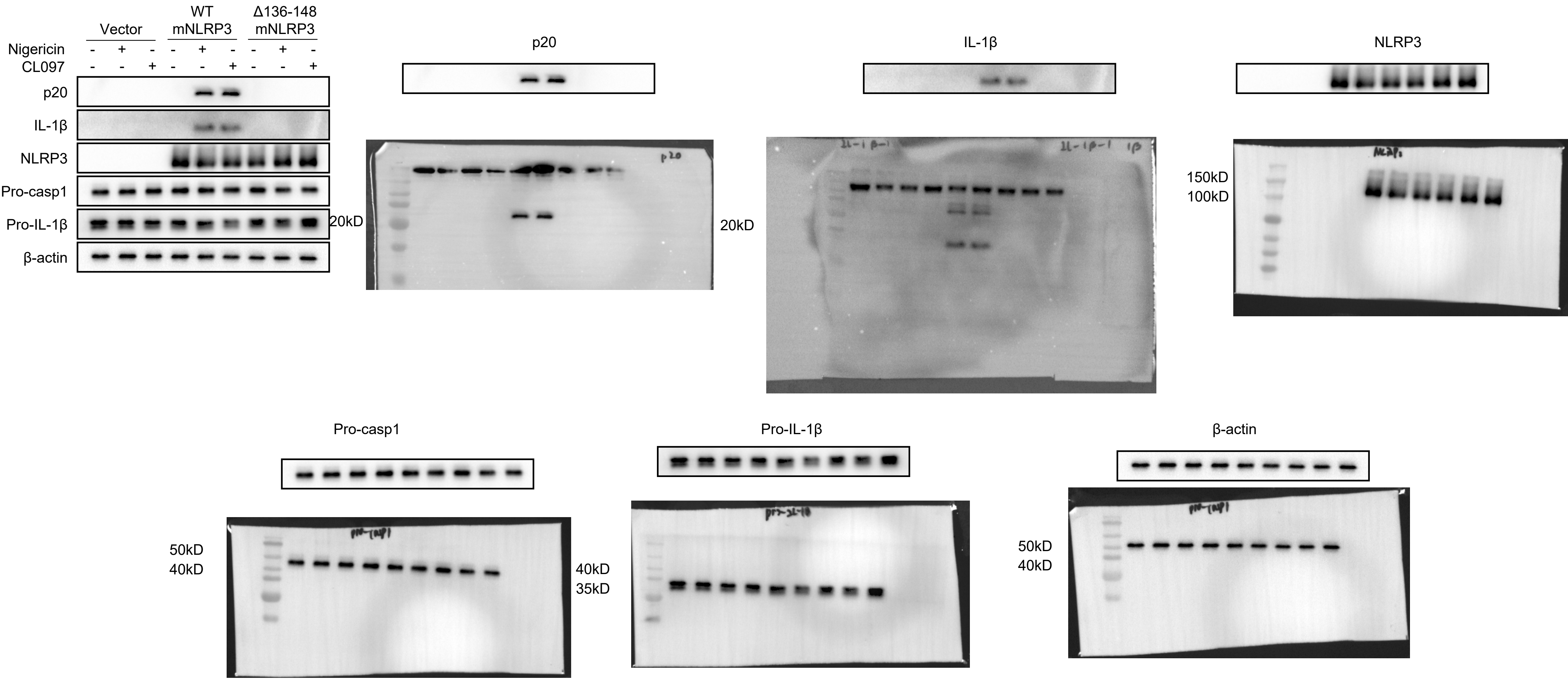
Vector
WT mNLRP3
Δ136-148 mNLRP3
Nigericin - + - - + - - + -
CL097 - - + - - + - - +
p20
IL-1β
NLRP3
Pro-casp1
Pro-IL-1β
β-actin
p20
20kD
20kD
IL-1β
NLRP3
150kD
100kD
Pro-casp1
50kD
40kD
Pro-IL-1β
40kD
35kD
β-actin
50kD
40kD

Fig. 5H

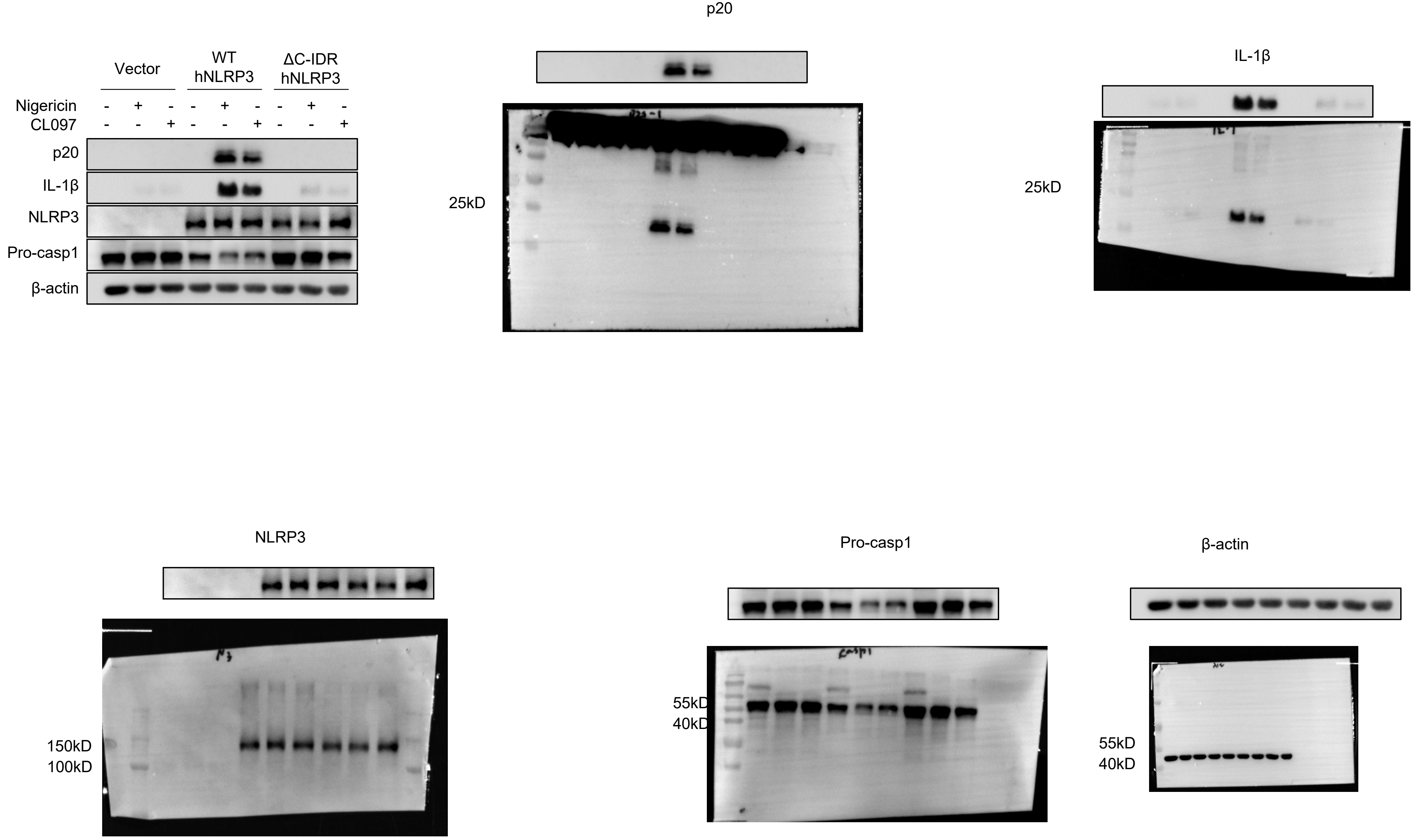

Vector
WT hNLRP3
ΔC-IDR hNLRP3
Nigericin - + - - + - - + -
CL097 - - + - - + - - +
p20
IL-1β
NLRP3
Pro-casp1
β-actin
p20
25kD
IL-1β
25kD
NLRP3
150kD
100kD
Pro-casp1
55kD
40kD
β-actin
55kD
40kD

Fig. 5J

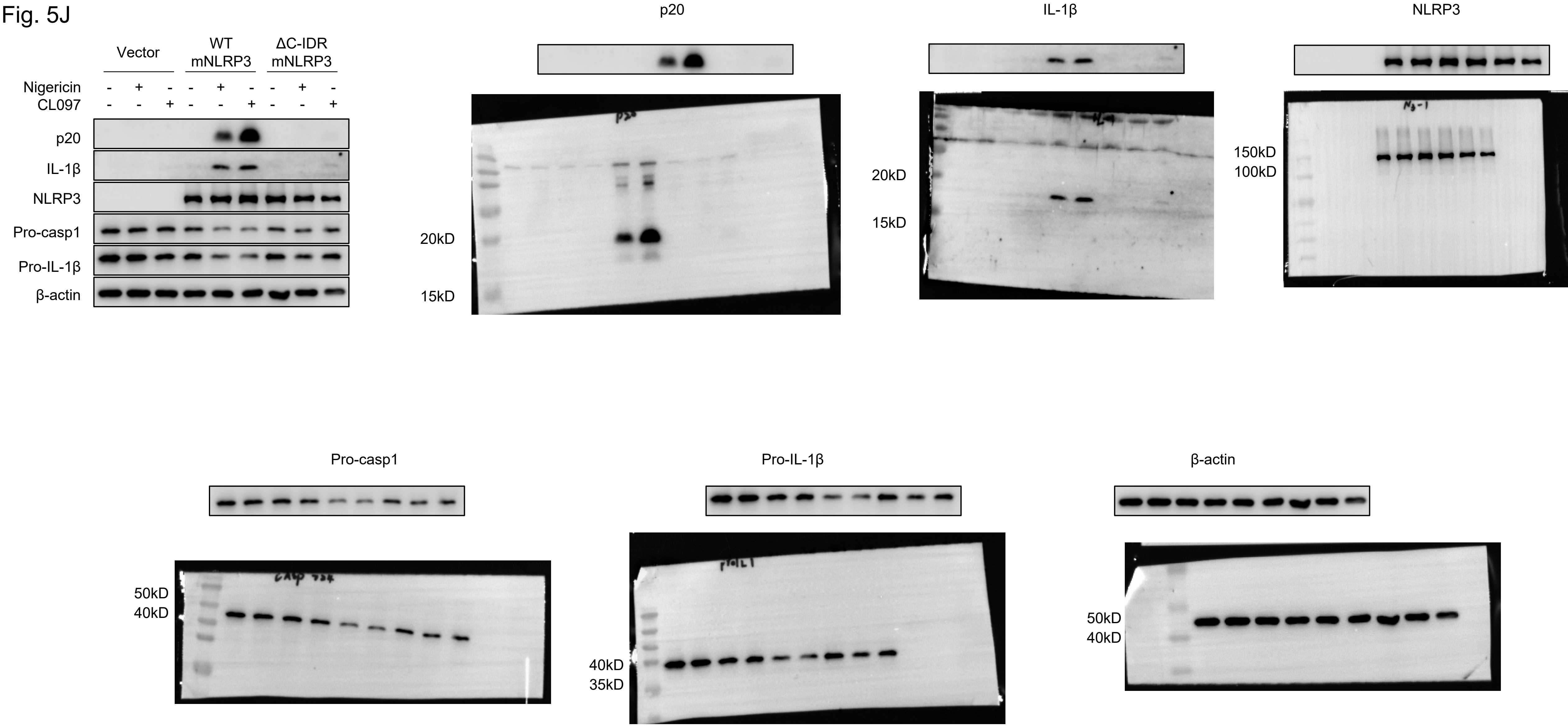
Vector
WT mNLRP3
ΔC-IDR mNLRP3
Nigericin - + - - + - - + -
CL097 - - + - - + - - +
p20
IL-1β
NLRP3
Pro-casp1
Pro-IL-1β
β-actin
p20
20kD
15kD
IL-1β
20kD
15kD
NLRP3
150kD
100kD
Pro-casp1
50kD
40kD
Pro-IL-1β
40kD
35kD
β-actin
50kD
40kD

Fig. S3F

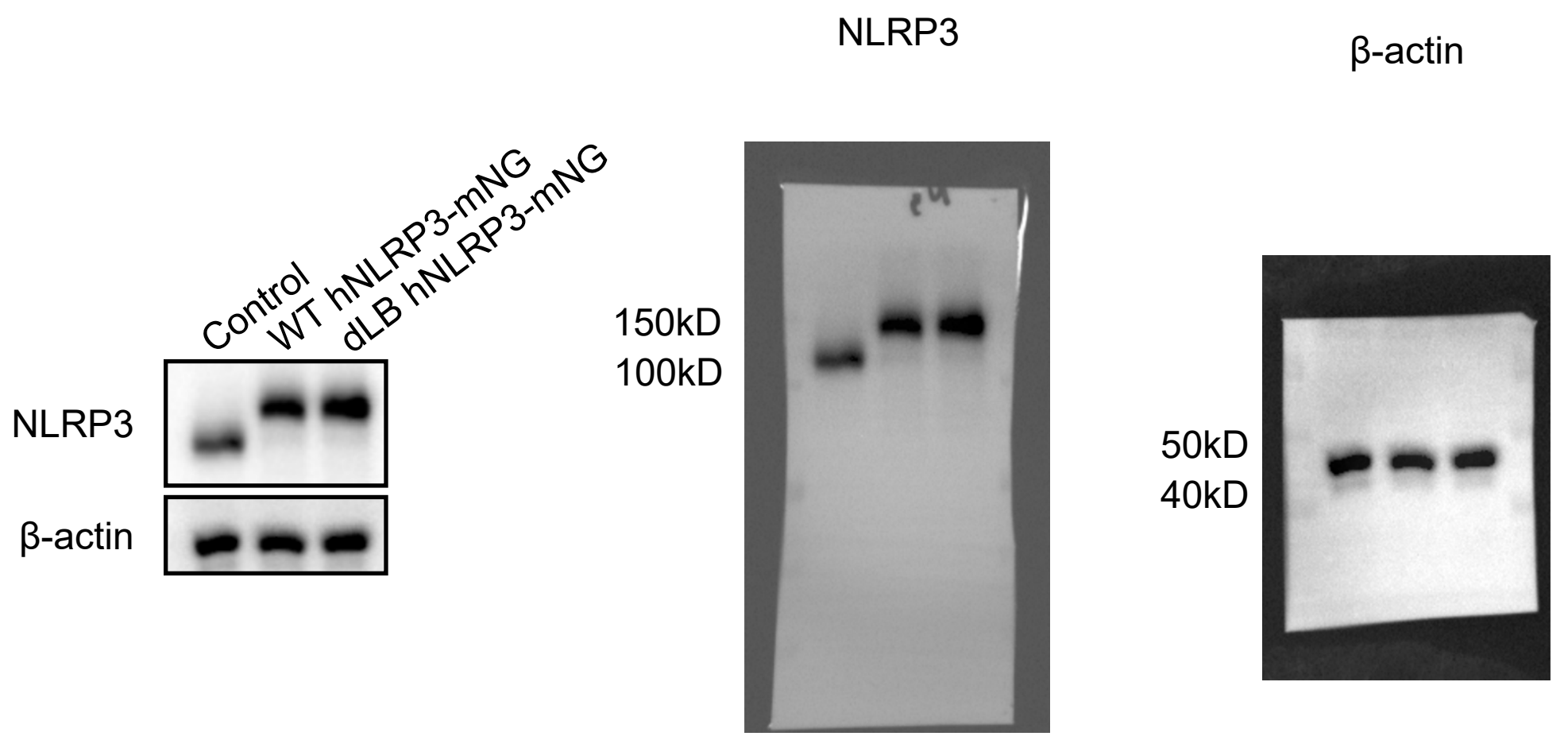

Fig. S5A

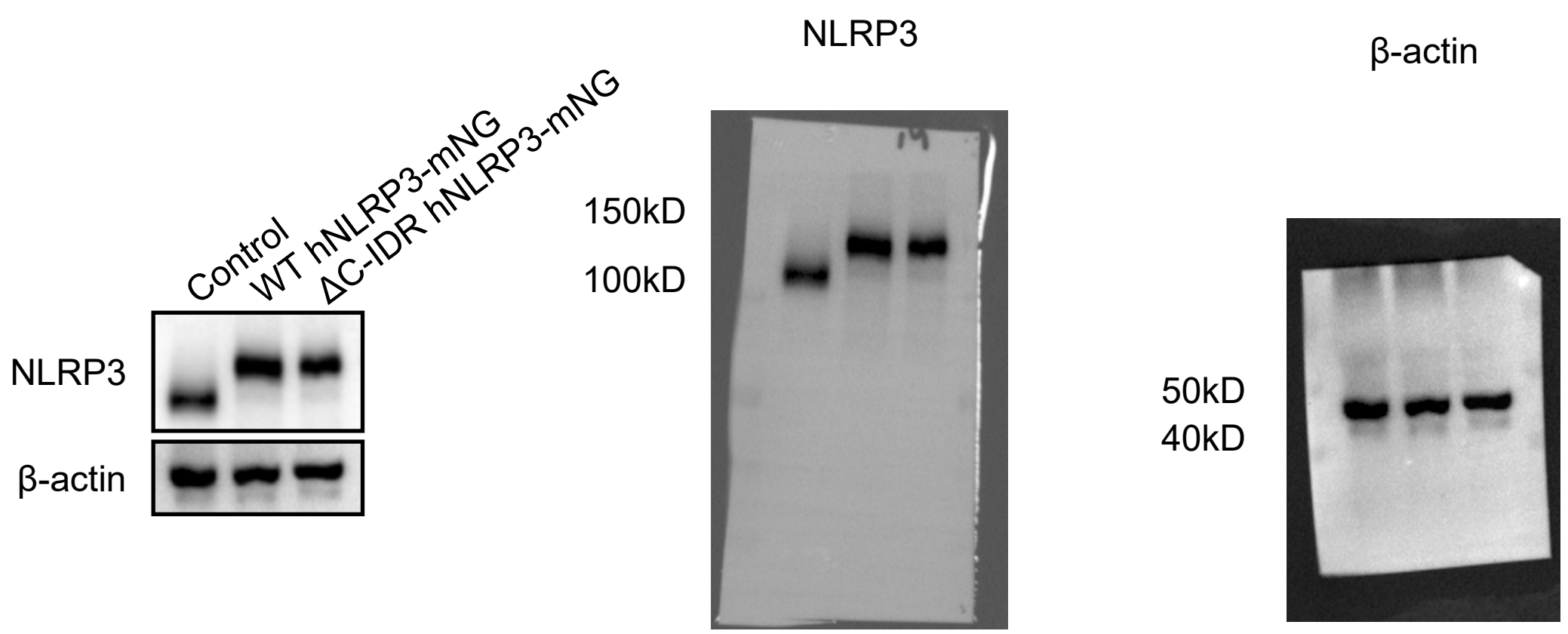

Fig. S3H

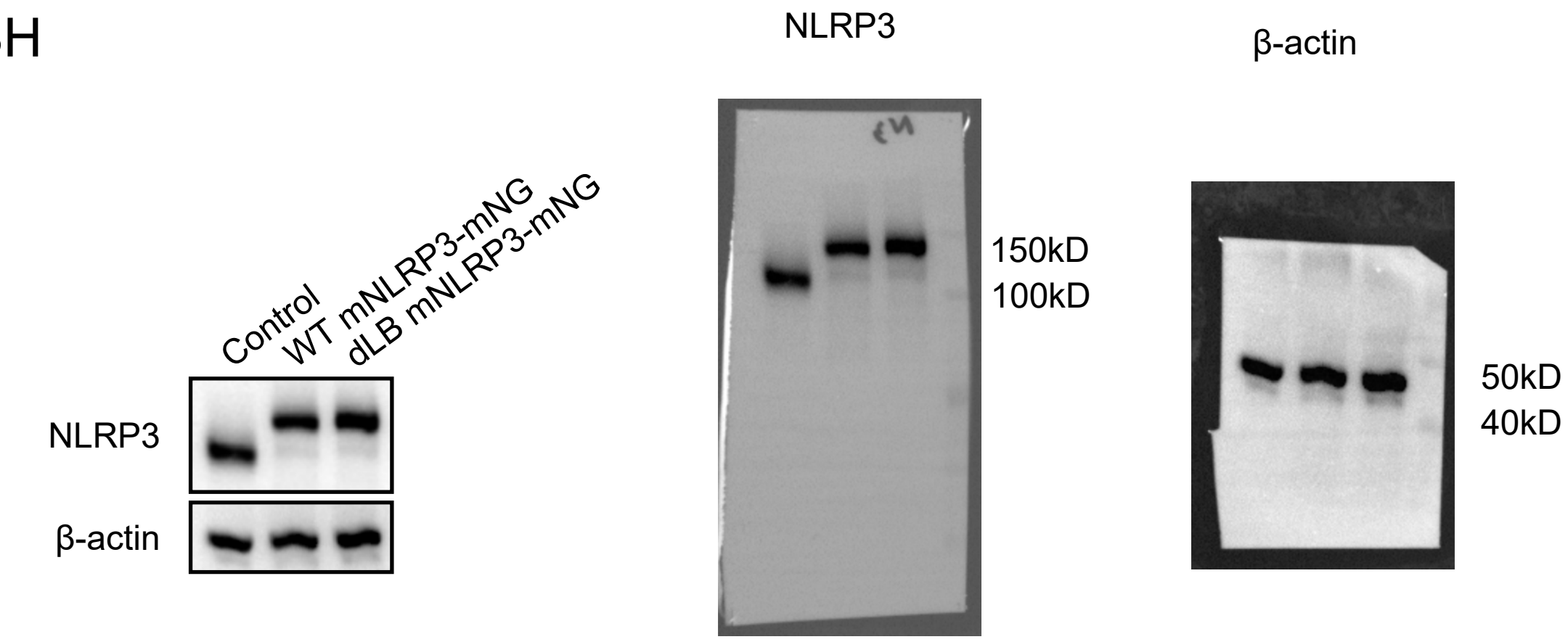

Fig. S5B

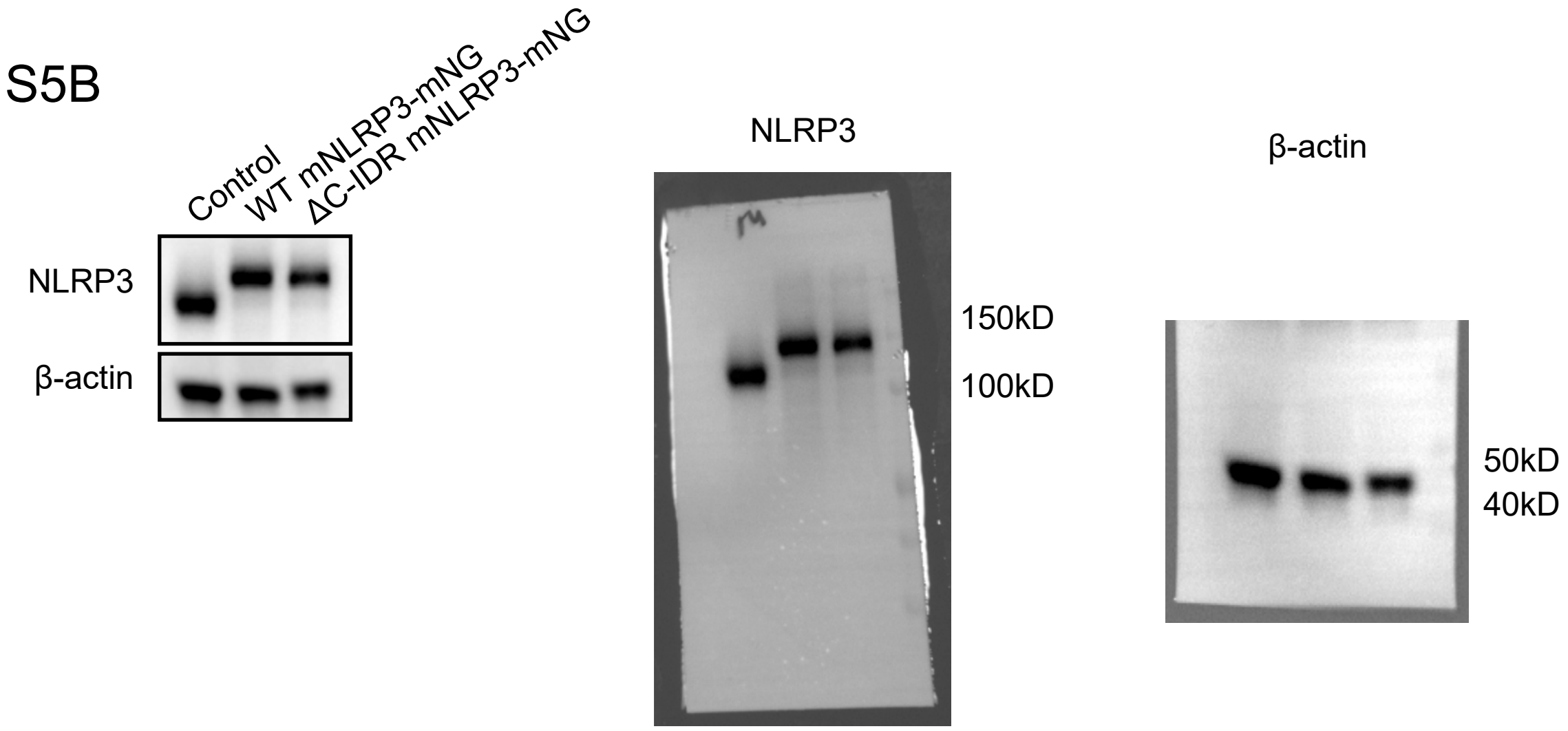

Supplement: Supplementary file 2 [file 41419_2026_9059_MOESM2_ESM.pdf]
