# Supplementary material for: Phase separation drives NLRP3 inflammasome activation independently of Golgi localization
Source: Cell Death Dis. 2026 Jul 6;17(1):839. doi: 10.1038/s41419-026-09059-4 (PMC13616974; doi:10.1038/s41419-026-09059-4)
Supplement: Supplementary file 3 [file 41419_2026_9059_MOESM6_ESM.pdf]

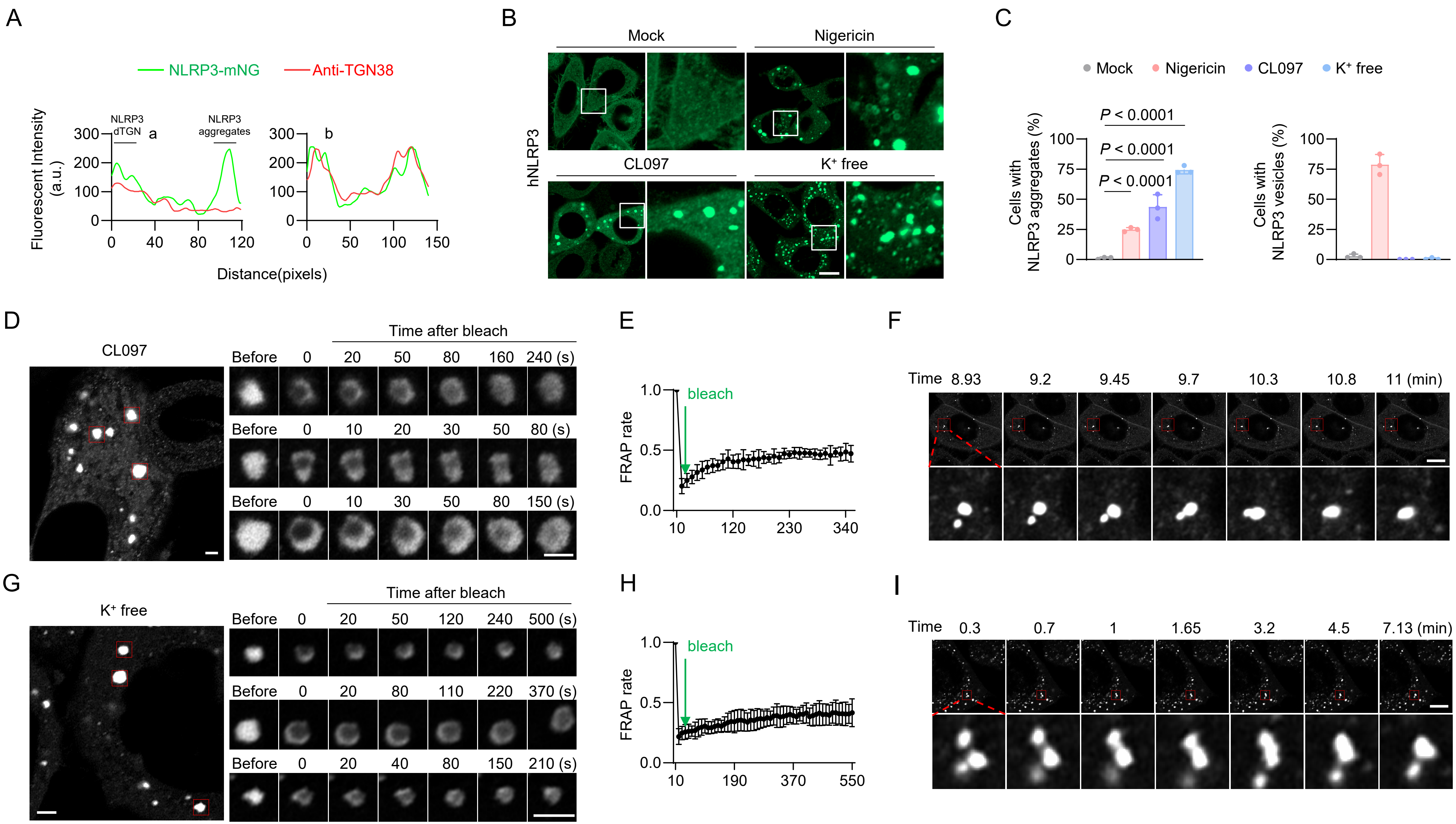
A
NLRP3-mNG
Anti-TGN38
Fluorescent Intensity (a.u.)
NLRP3 dTGN
a
NLRP3 aggregates
b
Distance(pixels)
B
Mock
Nigericin
hNLRP3
CL097
K⁺ free
C
Mock
Nigericin
CL097
K⁺ free
Cells with NLRP3 aggregates (%)
P < 0.0001
P < 0.0001
P < 0.0001
Cells with NLRP3 vesicles (%)
D
CL097
Time after bleach
Before
0
20
50
80
160
240 (s)
Before
0
10
20
30
50
80 (s)
Before
0
10
30
50
80
150 (s)
E
FRAP rate
bleach
F
Time
8.93
9.2
9.45
9.7
10.3
10.8
11 (min)
G
K⁺ free
Time after bleach
Before
0
20
50
120
240
500 (s)
Before
0
20
80
110
220
370 (s)
Before
0
20
40
80
150
210 (s)
H
FRAP rate
bleach
I
Time
0.3
0.7
1
1.65
3.2
4.5
7.13 (min)
