# Supplementary material for: Phase separation drives NLRP3 inflammasome activation independently of Golgi localization
Source: Cell Death Dis. 2026 Jul 6;17(1):839. doi: 10.1038/s41419-026-09059-4 (PMC13616974; doi:10.1038/s41419-026-09059-4)
Supplement: Supplementary file 4 [file 41419_2026_9059_MOESM7_ESM.pdf]

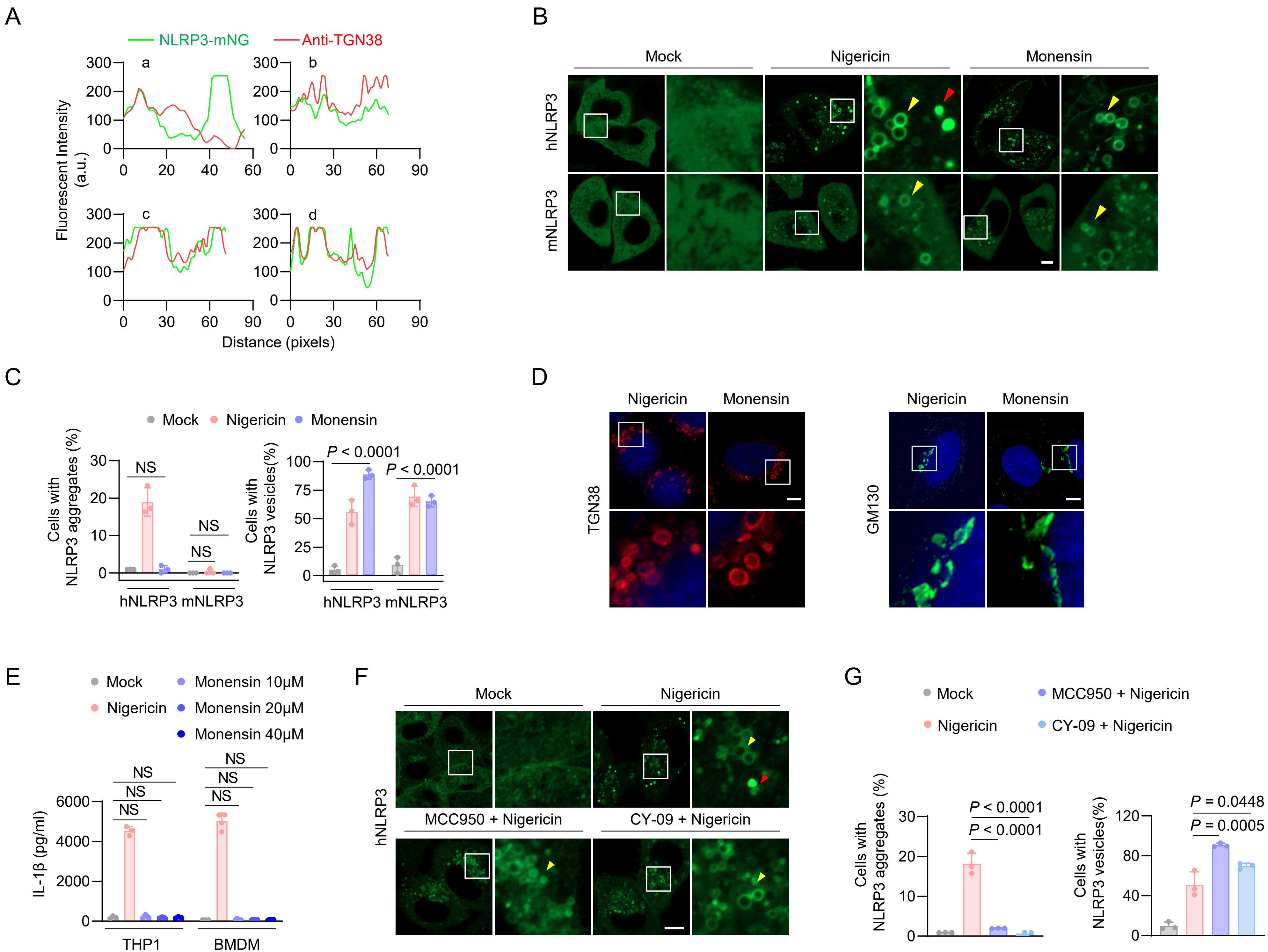

A
NLRP3-mNG
Anti-TGN38
Fluorescent Intensity (a.u.)
Distance (pixels)
a
b
c
d
B
Mock
Nigericin
Monensin
hNLRP3
mNLRP3
C
Mock
Nigericin
Monensin
Cells with NLRP3 aggregates (%)
Cells with NLRP3 vesicles(%)
NS
P < 0.0001
hNLRP3
mNLRP3
D
Nigericin
Monensin
TGN38
GM130
E
Mock
Nigericin
Monensin 10μM
Monensin 20μM
Monensin 40μM
IL-1β (pg/ml)
NS
THP1
BMDM
F
Mock
Nigericin
MCC950 + Nigericin
CY-09 + Nigericin
hNLRP3
G
Mock
Nigericin
MCC950 + Nigericin
CY-09 + Nigericin
Cells with NLRP3 aggregates (%)
Cells with NLRP3 vesicles(%)
P < 0.0001
P = 0.0448
P = 0.0005
