# Supplementary material for: Phase separation drives NLRP3 inflammasome activation independently of Golgi localization
Source: Cell Death Dis. 2026 Jul 6;17(1):839. doi: 10.1038/s41419-026-09059-4 (PMC13616974; doi:10.1038/s41419-026-09059-4)
Supplement: Supplementary file 5 [file 41419_2026_9059_MOESM8_ESM.pdf]

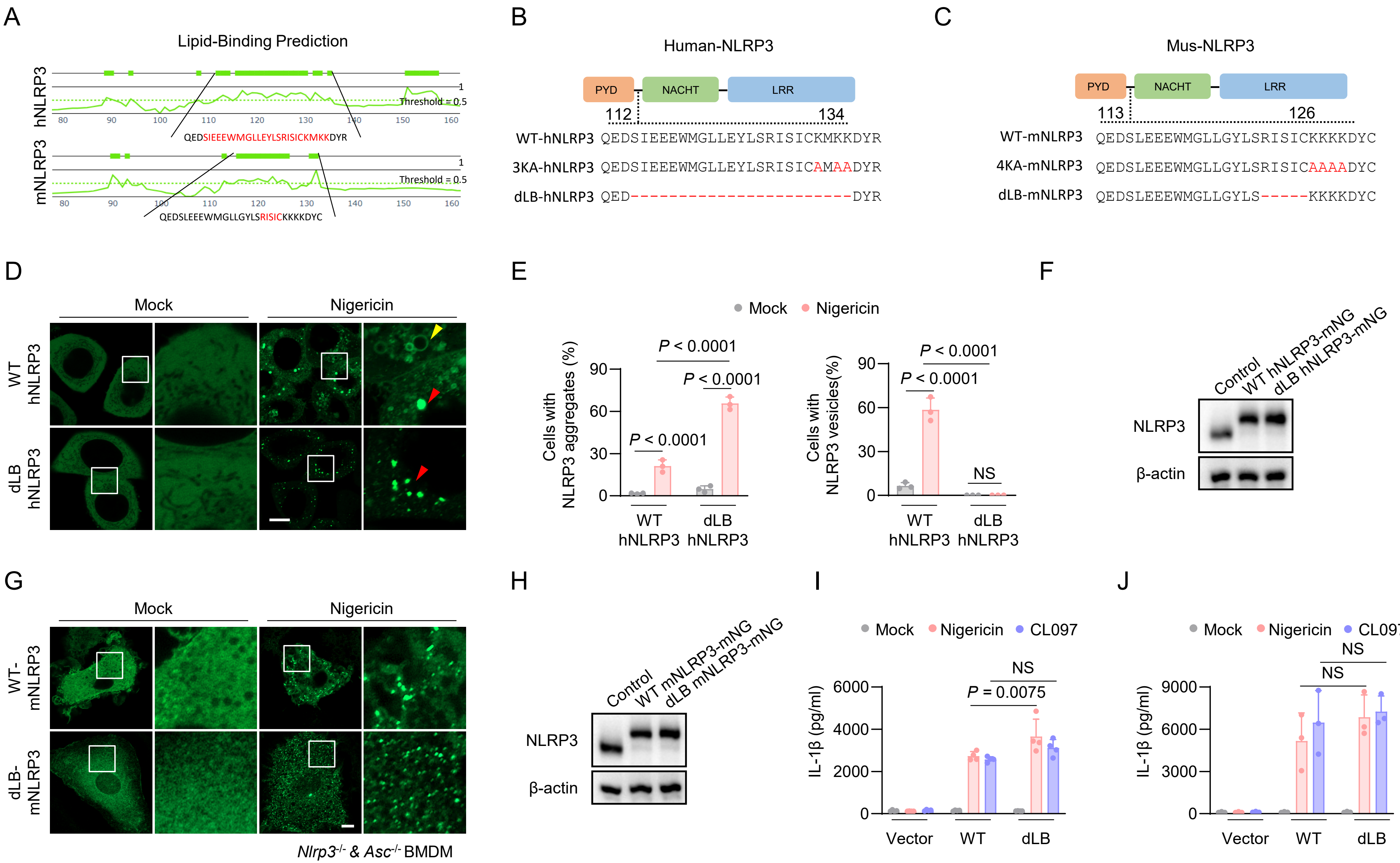
A
Lipid-Binding Prediction
hNLRP3
mNLRP3
Threshold = 0.5
QEDSIEEEWMGLLEYLSRISICKMKKDYR
QEDSLEEEWMGLLGYLSRISICKKKKDYC
B
Human-NLRP3
PYD
NACHT
LRR
112
134
WT-hNLRP3 QEDSIEEEWMGLLEYLSRISICKMKKDYR
3KA-hNLRP3 QEDSIEEEWMGLLEYLSRISICAMAADYR
dLB-hNLRP3 QED-----------------------DYR
C
Mus-NLRP3
113
126
WT-mNLRP3 QEDSLEEEWMGLLGYLSRISICKKKKDYC
4KA-mNLRP3 QEDSLEEEWMGLLGYLSRISICAAAADYC
dLB-mNLRP3 QEDSLEEEWMGLLGYLS-----KKKKDYC
D
Mock
Nigericin
WT hNLRP3
dLB hNLRP3
E
Mock
Nigericin
Cells with NLRP3 aggregates (%)
Cells with NLRP3 vesicles(%)
P < 0.0001
NS
WT hNLRP3
dLB hNLRP3
F
Control
WT hNLRP3-mNG
dLB hNLRP3-mNG
NLRP3
β-actin
G
WT-mNLRP3
dLB-mNLRP3
Nlrp3-/- & Asc-/- BMDM
H
Control
WT mNLRP3-mNG
dLB mNLRP3-mNG
I
Mock
Nigericin
CL097
IL-1β (pg/ml)
P = 0.0075
Vector
WT
dLB
J
