# Supplementary material for: Phase separation drives NLRP3 inflammasome activation independently of Golgi localization
Source: Cell Death Dis. 2026 Jul 6;17(1):839. doi: 10.1038/s41419-026-09059-4 (PMC13616974; doi:10.1038/s41419-026-09059-4)
Supplement: Supplementary file 6 [file 41419_2026_9059_MOESM9_ESM.pdf]

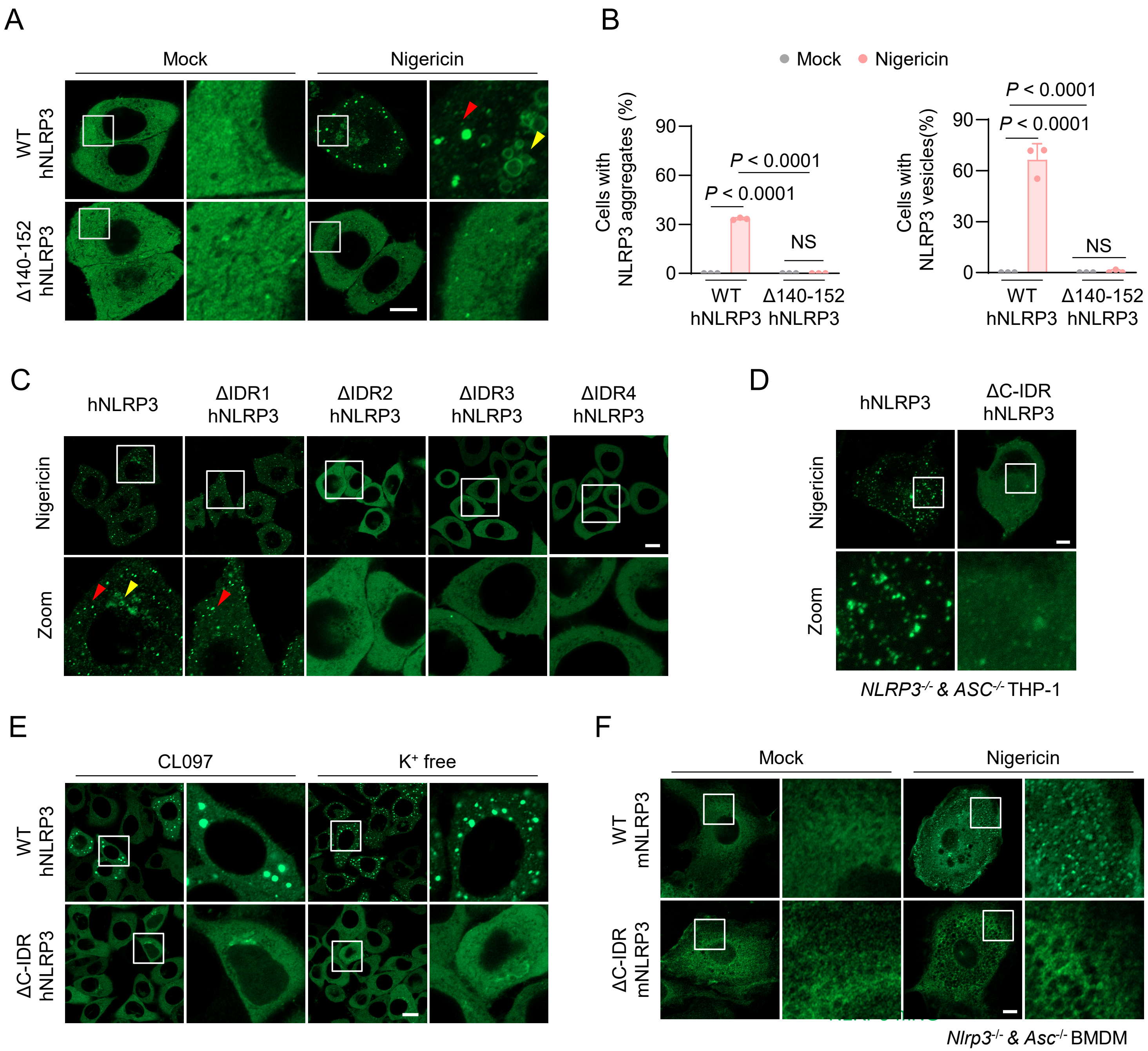

A
Mock
Nigericin
WT hNLRP3
Δ140-152 hNLRP3
B
Mock
Nigericin
Cells with NLRP3 aggregates (%)
90
60
30
0
P < 0.0001
P < 0.0001
NS
WT hNLRP3
Δ140-152 hNLRP3
Cells with NLRP3 vesicles(%)
P < 0.0001
P < 0.0001
NS
C
hNLRP3
ΔIDR1 hNLRP3
ΔIDR2 hNLRP3
ΔIDR3 hNLRP3
ΔIDR4 hNLRP3
Nigericin
Zoom
D
hNLRP3
ΔC-IDR hNLRP3
Nigericin
Zoom
NLRP3-/- & ASC-/- THP-1
E
CL097
K+ free
WT hNLRP3
ΔC-IDR hNLRP3
F
Mock
Nigericin
WT mNLRP3
ΔC-IDR mNLRP3
Nlrp3-/- & Asc-/- BMDM
