# Supplementary material for: Phase separation drives NLRP3 inflammasome activation independently of Golgi localization
Source: Cell Death Dis. 2026 Jul 6;17(1):839. doi: 10.1038/s41419-026-09059-4 (PMC13616974; doi:10.1038/s41419-026-09059-4)
Supplement: Supplementary file 7 [file 41419_2026_9059_MOESM1_ESM.docx]

**Supplementary Figure Legends:**

**Fig. S1 NLRP3 exhibits both Golgi-localization and LLPS.**

**(A)** Line-scan profiles showing normalized MFI of NLRP3 and TGN signals from (**Fig. 1A**).

**(B)** Representative confocal images of HeLa cells stably expressing human NLRP3 stimulated with 10 μM nigericin, 40 μM CL097, or K^+^-free medium for 60 min. Scale bar, 10 μm.

**(C)** Quantification of cells containing NLRP3 vesicles or aggregates (>60 cells per group, *n* = 3).

**(D–E)** FRAP analysis of human NLRP3–mNG in HeLa cells after 60 min of CL097 stimulation. Bleached regions are indicated (red boxes). Scale bar, 2 μm.
**(F)** Time-lapse imaging of NLRP3 droplet fusion in HeLa cells 60 min after CL097 stimulation. Scale bar, 5 μm.
**(G–H)** FRAP analysis of human NLRP3–mNG in HeLa cells after 60 min of K^+^-free medium treatment. Bleached regions are indicated (red boxes). Scale bar, 2 μm.
**(I)** Time-lapse imaging of NLRP3 droplet fusion in HeLa cells 60 min after K^+^-free medium treatment. Scale bar, 5 μm.

Data are presented as mean ± SD. In all quantifications, *n* represents the number of independent experiments. Data shown in (B, D, F, G, I) are representative of three independent experiments. Images in (B) are quantified in (C). Significance determined by one-way ANOVA followed by Tukey's multiple comparisons test (C).

**Fig. S2** **NLRP3 phase separation, but not Golgi localization, parallels inflammasome activation**

**(A)** Line-scan profiles showing normalized MFI of NLRP3 and TGN signals from (**Fig. 3A**).

**(B)** Representative confocal images of HeLa cells stably expressing wild-type human or mouse NLRP3 before and after stimulation with 10 μM nigericin or 10 μM monensin for 60 min. Scale bar, 5 μm.

**(C)** Quantification of cells containing NLRP3 vesicles or aggregates (>60 cells per group, *n* = 3).

**(D)** Representative confocal images showing the distribution of the cis-Golgi marker GM130 and the trans-Golgi network (TGN) marker TGN38 in HeLa cells. Cells were either left untreated or stimulated with 10 μM nigericin or 10 μM monensin for 1 h. Scale bar, 5 μm.
**(E)** IL-1β levels in supernatants from experiments shown in **(Fig. 3B–C)**, measured by ELISA (*n* = 3).

**(F)** Representative confocal images of HeLa cells stably expressing human NLRP3 treated for 60 min with 10 μM nigericin alone, 5 μM MCC950 and 10 μM nigericin, or 20 μM CY-09 and 10 μM nigericin. Scale bar: 10 μm.

**(G)** Quantification of cells containing NLRP3 vesicles or aggregates (>60 cells per group, *n* = 3).

Data are presented as mean ± SD. In all quantifications, *n* represents the number of independent experiments. Data shown in (B, D, F) are representative of three independent experiments. Images in (B, F) are quantified in (C, G) respectively. Significance determined by two-way ANOVA (C) or one-way ANOVA (E, G) followed by Tukey's multiple comparisons test; NS, not significant.

**Fig. S3 dLB-NLRP3 loses TGN localization but retains inflammasome activation.**

**(A)** Schematic diagram of lipid binding region prediction of human and mouse NLRP3.

**(B, C)** Schematic diagram of different mutations of human or mouse NLRP3. dLB: deletion of lipid binding region.

**(D)** Representative images of HeLa cells stably expressing wild-type or dLB mutant human NLRP3 before and after 10 μM nigericin stimulation for 60 min. Scale bar, 10 μm.

**(E)** Quantification of cells containing NLRP3 vesicles or aggregates (>60 cells per group, *n* = 3).
**(F)** Quantitative immunoblot analysis comparing the expression levels of reconstituted wild-type and dLB mutant human NLRP3-mNG constructs in *NLRP3*^-/-^ THP-1 cells to endogenous NLRP3 in wild-type THP-1 cells. Before analysis, THP-1 cells were differentiated with PMA for 24 h and subsequently cultured in fresh medium for 48 h.

**(G)** Representative images of stably expressing wild-type or dLB mutant mouse NLRP3 in *Nlrp3^-^*^/-^ and *Asc*^-/-^ double-knockout BMDMs treated with 10 μM nigericin (60 min). Scale bar, 5 μm.

**(H)** Quantitative immunoblot analysis comparing the expression levels of reconstituted wild-type and dLB mutant mouse NLRP3-mNG constructs in *Nlrp3*^-/-^ BMDMs to endogenous NLRP3 in wild-type BMDMs. Before analysis, BMDMs were primed with 50 ng/mL LPS for 6 h.

**(I)** IL-1β levels in supernatants from experiments shown in **(Fig. 3G)**, measured by ELISA (*n* = 3).

**(J)** IL-1β levels in supernatants from experiments shown in **(Fig. 3K)**, measured by ELISA (*n* = 3).

Data are presented as mean ± SD. In all quantifications, *n* represents the number of independent experiments. Data shown in (D, F, G, H) are representative of three independent experiments. Images in (D) are quantified in (E). Significance determined by two-way ANOVA followed by Tukey's multiple comparisons test (E, I, J); NS, not significant.

**Fig. S4 ΔC-IDR NLRP3 abolishes phase separation.**

**(A)** Representative images of HeLa cells stably expressing wild-type or Δ140-152 mutant human NLRP3 before and after 10 μM nigericin stimulation for 60 min. Scale bar, 10 μm.

**(B)** Quantification of cells containing NLRP3 vesicles or aggregates (>60 cells per group, *n* = 3).

**(C)** Representative images of HeLa cells stably expressing human NLRP3, ΔIDR1 (Δ95-109) NLRP3, ΔIDR2 (Δ422-437) NLRP3, ΔIDR3 (Δ541-556) NLRP3, and ΔIDR4 (Δ738-752) NLRP3. Cells were stimulated with 10μM nigericin for 90 min. Scale bar, 10 μm.

**(D)** Representative images of stably expressing wild-type or ΔC-IDR mutant human NLRP3 in *NLRP3^-^*^/-^ and *ASC*^-/-^ double-knockout THP-1 cells were stimulated with 10 μM nigericin (60 min). Scale bar, 5 μm.

**(E)** Representative images of human NLRP3 or ΔIDR human NLRP3 stably expressing HeLa cells were stimulated with 30 μM CL097 for 60 min or K^+^-free treated for 60 min. Scale bar, 10 μm.

**(F)** Representative images of stably expressing wild-type or ΔC-IDR mutant mouse NLRP3 in *Nlrp3^-^*^/-^ and *Asc*^-/-^ double-knockout BMDMs treated with 10 μM nigericin (60 min). Scale bar, 5 μm.

Data are presented as mean ± SD. In all quantifications, *n* represents the number of independent experiments. Data shown in (A, C, D, E, F) are representative of three independent experiments. Images in (A) are quantified in (B). Significance determined by two-way ANOVA followed by Tukey's multiple comparisons test (B); NS, not significant.

**Fig. S5 Supplementary functional validations of NLRP3 mutants.**

**(A)** Quantitative immunoblot analysis comparing the expression levels of reconstituted wild-type and ΔC-IDR mutant human NLRP3-mNG constructs in *NLRP3*^-/-^ THP-1 cells to endogenous NLRP3 in wild-type THP-1 cells. Before analysis, THP-1 cells were differentiated with PMA for 24 h and subsequently cultured in fresh medium for 48 h.

**(B)** Quantitative immunoblot analysis comparing the expression levels of reconstituted wild-type and ΔC-IDR mutant mouse NLRP3-mNG constructs in *Nlrp3*^-/-^BMDMs to endogenous NLRP3 in wild-type BMDMs. Before analysis, BMDMs were primed with 50 ng/mL LPS for 6 h.

**(C)** Representative confocal images showing the phase separation behaviors of wild-type NLRP3 and the finer mapping mutant at the R972-K973 motif (RK-AA) within the C-terminal IDR in HeLa cells stimulated with 10 μM nigericin for 60 min. Scale bar, 5 μm.

**(D)** Representative confocal images showing NLRP3 aggregation in *NLRP3^-/-^* and *ASC^-/-^* double-knockout THP-1 cells stably expressing WT or mutant NLRP3. Cells were stimulated with 500 ng/mL LPS for 12 h. Scale bar, 20 μm.

**(E)** ELISA quantification of IL-1β secretion from the supernatants of *NLRP3^-/-^* THP-1 cells stably expressing WT or mutant NLRP3, following stimulation with 500 ng/mL LPS for 12 h (*n* = 3).

**(F)** Representative confocal images of ASC speck formation in *Nlrp3^-/-^* and *Asc^-/-^* double-knockout BMDMs reconstituted with WT or mutant NLRP3. Cells were primed with LPS (50 ng/mL for 3h) and then stimulated with 10 μM nigericin for 15 min. Scale bar, 20 μm.

**(G)** Percentage of ASC speck-positive cells from the experiments shown in (F) (>50 cells per group, *n* = 3).

Data are presented as mean ± SD. In all quantifications, *n* represents the number of independent experiments. Data shown in (A, B, C, D, F) are representative of two or three independent experiments. Images in (F) are quantified in (G). Significance determined by one-way ANOVA followed by Tukey's multiple comparisons test (E, G); NS, not significant.

**Supplementary Movie Legends:**

**Movie S1. Nigericin-Induced NLRP3 Droplet Formation (Related to Fig. 1J)**

Time-lapse micrographs of human NLRP3 droplet fusion in HeLa cells after 60 min of nigericin stimulation.

**Movie S2. CL097-Induced NLRP3 Droplet Formation (Related to Fig. S1F)**

Time-lapse micrographs of human NLRP3 droplet fusion in HeLa cells after 60 min of CL097 stimulation.

**Movie S3. K^+^-free Medium–Induced NLRP3 Droplet Formation (Related to Fig. S1I)**

Time-lapse micrographs of human NLRP3 droplet fusion in HeLa cells after K^+^-free treatment for 60 min.

**Original data**

Full and uncropped western blots are provided in the Supplementary Material.
